# Assessing the impact of different contact patterns on disease transmission: Taking COVID-19 as a case

**DOI:** 10.1371/journal.pone.0300884

**Published:** 2024-04-11

**Authors:** Fenfen Zhang, Juan Zhang, Mingtao Li, Zhen Jin, Yuqi Wen

**Affiliations:** 1 College of Mathematics and Statistics, Taiyuan Normal University, Jinzhong, Shanxi, China; 2 Shanxi College of Technology, Shuozhou, Shanxi, China; 3 Complex Systems Research Center, Shanxi University, Taiyuan, Shanxi, China; 4 Shanxi Key Laboratory of Mathematical Techniques and Big Data Analysis on Disease Control and Prevention, Shanxi University, Taiyuan, Shanxi, China; 5 Key Laboratory of Complex Systems and Data Science of Ministry of Education, Shanxi University, Taiyuan, Shanxi, China; 6 School of Mathematics, Taiyuan University of Technology, Taiyuan, Shanxi, China; 7 School of Materials Science & Engineering, Beijing Institute of Technology, Beijing, China; East China Normal University, CHINA

## Abstract

Human-to-human contact plays a leading role in the transmission of infectious diseases, and the contact pattern between individuals has an important influence on the intensity and trend of disease transmission. In this paper, we define regular contacts and random contacts. Then, taking the COVID-19 outbreak in Yangzhou City, China as an example, we consider age heterogeneity, household structure and two contact patterns to establish discrete dynamic models with switching between daytime and nighttime to depict the transmission mechanism of COVID-19 in population. We studied the changes in the reproduction number with different age groups and household sizes at different stages. The effects of the proportion of two contacts patterns on reproduction number were also studied. Furthermore, taking the final size, the peak value of infected individuals in community and the peak value of quarantine infected individuals and nucleic acid test positive individuals as indicators, we evaluate the impact of the number of random contacts, the duration of the free transmission stage and summer vacation on the spread of the disease. The results show that a series of prevention and control measures taken by the Chinese government in response to the epidemic situation are reasonable and effective, and the young and middle-aged adults (aged 18-59) with household size of 6 have the strongest transmission ability. In addition, the results also indicate that increasing the proportion of random contact is beneficial to the control of the infectious disease in the phase with interventions. This work enriches the content of infectious disease modeling and provides theoretical guidance for the prevention and control of follow-up major infectious diseases.

## Introduction

Contact transmission is the main route for infectious diseases. For many infectious diseases, such as SARS in 2003, MERS in 2012 and COVID-19 in 2019, there are no specific drugs in the early stages of the outbreak [1–6], and disease prevention and control relies on non-pharmaceutical interventions (NPIs), and the key point of NPIs is to change human daily contact behavior. A major challenge is to identify and quantify the human contact behavior on the transmission of infectious diseases. And the quantitative understanding of the human contact behavior is useful to accurately predict of new or re-emerging infectious diseases and improve targeting of prevention and control interventions.

There are lots of meaningful works about the effect of human contact behavior on disease transmission. Eames established a SIR mathematical model combining pair approximation with mean field to study the effects of regular and random contacts on disease transmission [7]. Volz et al. constructed a clustering random network to study the effects of heterogeneous and aggregated contact patterns on epidemic dynamics [8]. Zhang et al. studied the impact of changes in contact patterns on the spread of COVID-19 in China [9]. Liu et al. established a multi-layer contact network based on detailed socio-demographic data to simulate the spread of disease, and studied the impact of heterogeneity and aggregation of human interaction on basic epidemiological indicators [10]. Jarvis et al. used questionnaires to compare changes in contact patterns before and after the “lockdown”, and then quantified the impact of physical distance policy on COVID-19 transmission in the United States [11]. These results reflect the important role of human contacts in disease transmission from different aspects. However, none of these studies took into account changes in the regularity of contact patterns from day to day.

The actual contact patterns may be more complex and will differ between daytime and nighttime. During the daytime people will go to workplaces or school, or stay at home or go somewhere else, and come back to residence at night to rest. Moreover, we define the contacts between families, classmates, classmates and teachers at school, between colleagues in workplaces, and between elderly people in the fixed activity places as regular contacts. In addition to regular contacts, people will also come into contact with some others for a short time at short distances in some leisure and entertainment places or on their way to work/school or on their way home. Such contacts are defined as random contacts. Contacts that occur during daytime include regular contacts and random contacts, and the proportion and the number of regular and random contacts depends on age. Contacts that occur at the residence at night are regular contacts, and the number of contacts depends on household size. Specifically, if you are between 18 and 59 years old and have a job, you will have regular contacts with your colleagues during daytime. If you are older than 60, then you may stay at home alone, you may participate in activities and have regular contacts with the elderly, or you may have regular contacts with your peers in the same household or minors during daytime. If you household size is 2, then you will have regular contact with one individual at home during nighttime. While, if you household size is 5, then you will have regular contacts with four individuals at home during nighttime. Therefore, considering the two contact patterns, the heterogeneity of age structure and household size has to be taken into account. There are a lot of related studies about the effect of age structure on disease transmission [12–15] or about the effect of household size on disease transmission [16, 17]. However, the effect of the interaction of age structure, household size, regular and random contacts remains unclear. In order to fill this gap, taking the COVID-19 outbreak in Yangzhou City, China as an example, we categorize the study population by age structure and household size, and take into account different contact patterns during daytime and nighttime, and then establish discrete dynamic models with switching between daytime and nighttime to assess the impact of these factors in disease transmission.

The paper is organized as follows. In the Model formulation section, two discrete dynamic models with Markov properties are developed based on COVID-19 transmission mechanism and whether to take the NPIs. In the Model analysis section, a simple analysis of the model is performed and the expressions for the basic reproduction number and control reproduction number are given. In the Model application section, the models are applied to the outbreak of COVID-19 in Yangzhou City in July 2021. Then, the least square (LS) method is used to fit the model with the nucleic acid test positive cases of Yangzhou City from July 28th to September 2ed, 2021. The impact of age heterogeneity, household structure and two contact patterns on disease transmission is then analyzed by applying numerical simulation of model. The Conclusion and discussion section gives the conclusion and discussion of this work.

## Model formulation

The total number of the population is recorded as *N*. And the population is divided into *K* groups according to the household size, where *N*_*k*_ (*k* = 1, 2, ⋯, *K*) represents the total number of individuals belonging to households with size *k*. In addition, the population is divided into *A* groups according to age, and $N^{a_{i}}$ (*i* = 1, 2, ⋯, *A*) represents the number of the individuals whose age are grouped into *a*_*i*_. $N_{k}^{a_{i}}$ represents the number of the individuals with household size-*k*, age group-*a*_*i*_. Then, we have:

$$\sum_{k=1}^{K}N_{k}^{a_{i}}=N^{a_{i}},\sum_{i=1}^{A}N_{k}^{a_{i}}=N_{k},\sum_{k=1}^{K}\sum_{i=1}^{A}N_{k}^{a_{i}}=N.$$

Assume that daytime and nighttime transmissions last for 10 hours and 14 hours respectively. That is, the model uses the daytime equations for an interval of $\frac{5}{12}$ day, and then the nighttime equations are used for the subsequent $\frac{7}{12}$ day. Birth and death are disregarded. At time *t*, the individuals are divided into six categories according to the state of the disease: susceptible individuals (*S*(*t*)), exposed individuals who are infected but do not have infectivity (*E*(*t*)), infectious individuals (*I*(*t*)), recovered individuals (*R*(*t*)), quarantined exposed and infectious individuals who had been traced and quarantined as a close contact (*Q*(*t*), these individuals have not had nucleic acid test or nucleic acid test results are negative), nucleic acid test positive individuals (*H*(*t*)). It is assumed that quarantined individuals and nucleic acid test positive individuals are not involved in the spread of the disease. Recovered individuals develop natural immunity and will not be infected again during the study period. The number of susceptible individuals who are traced and quarantined is only a small part of the total population, and the infected individuals who have come into contact with these susceptible individuals have been quarantined and will not infect them in a short period of time, thus tracing and quarantine of these susceptible individuals has little impact on the spread of the disease. At the same time, in order to facilitate the analysis, the paper does not consider the tracing and quarantine of the susceptible individuals.

### The infection probability with regular contacts

During daytime, regular contacts we considered mainly refers to the contacts between families at home, or between classmates, classmates and teachers at school, between colleagues in the workplaces, or between the elderly people in the fixed activity places. Suppose that everyone has only one regular contact place during daytime, either home, school, workplace or activity place. For population with the same household size, same age group the number of regular contacts $C_{k}^{a_{i}}\left(t\right)$ is a random variable with independent and identically distributed if the regular contact places are same. And we use *r* (*r* = 1, 2, ⋯) to indicate the number of actual contacts. The susceptibility of a susceptible individual with age group *a*_*i*_ is recorded as $\lambda^{a_{i}}$. Thus, during daytime, for each susceptible individual with household size-*k*, age group-*a*_*i*_, the probability of being infected as a result of regular contacts is:

$$\alpha_{k}^{a_{i}}\left(t\right)=1-\sum_{\iota }P\left(L_{k}^{a_{i}}\left(t\right)=\iota \right)(\sum_{r}P\left(C_{k}^{a_{i}}\left(t\right)=r|L_{k}^{a_{i}}\left(t\right)=\iota \right)P_{S}\left(t\right)).$$

Where *ι* ∈ {*SC*, *HO*, *WO*, *AC*} (*SC* represents school, *HO* represents home, *WO* represents workplace, *AC* represents activity place), and $P(L_{k}^{a_{i}}\left(t\right)=\iota )$ represents the probability that the place of regular contacts of an individual with household size-*k*, age group-*a*_*i*_ is *ι*. $P(C_{k}^{a_{i}}\left(t\right)=r|L_{k}^{a_{i}}\left(t\right)=\iota )$ denotes the probability that the number of regular contacts is *r* under the condition that the place of regular contacts is *ι*. While $P_{S}\left(t\right)=P\left(\text{Uninfected}\;\text{during}\;\text{daytime}|L_{k}^{a_{i}}\left(t\right)=\iota ,C_{k}^{a_{i}}\left(t\right)=r\right)$ represents the probability that the individual is not infected during daytime under the condition that the place of regular contacts is *ι* and the number of regular contacts is *r*.

At night, individuals only have regular contacts with their families at home. Then, for each susceptible individual with household size-*k*, age group-*a*_*i*_, the probability of being infected as a result of contact with families is:

$$\sigma_{k}^{a_{i}}\left(t\right)=1-P\left(\text{Not}\;\text{infected}\;\text{by}\;\text{his}\;\text{family}\;\text{members}\right).$$

### The infection probability with random contacts

During daytime, for each susceptible individual with household size-*k*, age group-*a*_*i*_, the infection probability of being infected as a result of random contacts is:

$$\beta_{k}^{a_{i}}\left(t\right)=1-{\left(1-\lambda^{a_{i}}\frac{I(t)}{N}\right)}^{X_{k}^{a_{i}}\left(t\right)},$$

where *I*(*t*) represents the total number of infectious individuals in the population at time *t*, and $X_{k}^{a_{i}}\left(t\right)$ represents the number of random contacts at time *t*. $1-\lambda^{a_{i}}\frac{I(t)}{N}$ represents the probability of not being infected after contact with an infectious individual, and ${\left(1-\lambda^{a_{i}}\frac{I(t)}{N}\right)}^{X_{k}^{a_{i}}\left(t\right)}$ represents the probability of not being infected after contact with $X_{k}^{a_{i}}\left(t\right)$ random contacts.

**Fig 1:**
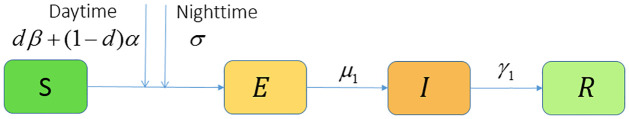
The flowchart of COVID-19 transmission during the free transmission stage.

Based on the disease transmission mechanism and whether to take NPIs, two discrete dynamic model models in finite state space are developed. The two models are switched at time *χ*, which is the time when NPIs start to implement. According to the free transmission stage, combined with the flow chart (Fig 1), the model (2.1) can be used to describe the transmission process of COVID-19:

$$\begin{array}{c} \left\{ \begin{array}{cc} S_{k}^{a_{i}}\left(t\right) & =\left(1-f_{k}^{a_{i}}\left(t-1\right)\right)S_{k}^{a_{i}}\left(t-1\right),\\ E_{k}^{a_{i}}\left(t\right) & =f_{k}^{a_{i}}\left(t-1\right)S_{k}^{a_{i}}\left(t-1\right)+\left(1-\mu_{1}\right)E_{k}^{a_{i}}\left(t-1\right),\\ I_{k}^{a_{i}}\left(t\right) & =\mu_{1}E_{k}^{a_{i}}\left(t-1\right)+\left(1-\gamma_{1}\right)I_{k}^{a_{i}}\left(t-1\right),\\ R_{k}^{a_{i}}\left(t\right) & =\gamma_{1}I_{k}^{a_{i}}\left(t-1\right)+R_{k}^{a_{i}}\left(t-1\right), \end{array}\right. \end{array}$$ (1)

Here, $S_{k}^{a_{i}}\left(t\right)\left(E_{k}^{a_{i}}\left(t\right),I_{k}^{a_{i}}\left(t\right),R_{k}^{a_{i}}\left(t\right)\right)$ represents the number of susceptible (exposed, infectious, recovered) individuals at time *t* with household size-*k*, age group-*a*_*i*_. $f_{k}^{a_{i}}\left(t\right)$ represents the probability of being infected of a susceptible individual with household size-*k*, age group-*a*_*i*_, and

$$f_{k}^{a_{i}}\left(t\right)=\{\begin{array}{c} d^{a_{i}}\beta_{k}^{a_{i}}\left(t\right)+\left(1-d^{a_{i}}\right)\alpha_{k}^{a_{i}}\left(t\right),\text{daytime},\\ \sigma_{k}^{a_{i}}\left(t\right),\text{nighttime}. \end{array}$$

Here, $d^{a_{i}}$ is the proportion of random contacts which only depends on age, and $1-d^{a_{i}}$ is the proportion of regular contacts. *μ*_1_ is the rate of $E_{k}^{a_{i}}\to I_{k}^{a_{i}}$, *γ*_1_ is recovery rate of infectious individuals. In the first equation of system (1), $f_{k}^{a_{i}}\left(t-1\right)S_{k}^{a_{i}}\left(t-1\right)$ represents the reduction in the number of susceptible individuals due to infection. In the second equation of system (1), $\mu_{1}^{a_{i}}\left(t-1\right)E_{k}^{a_{i}}\left(t-1\right)$ represents the decrease of exposed individuals after the incubation period. The implications of the other equations can be obtained in a similar way.

**Fig 2:**
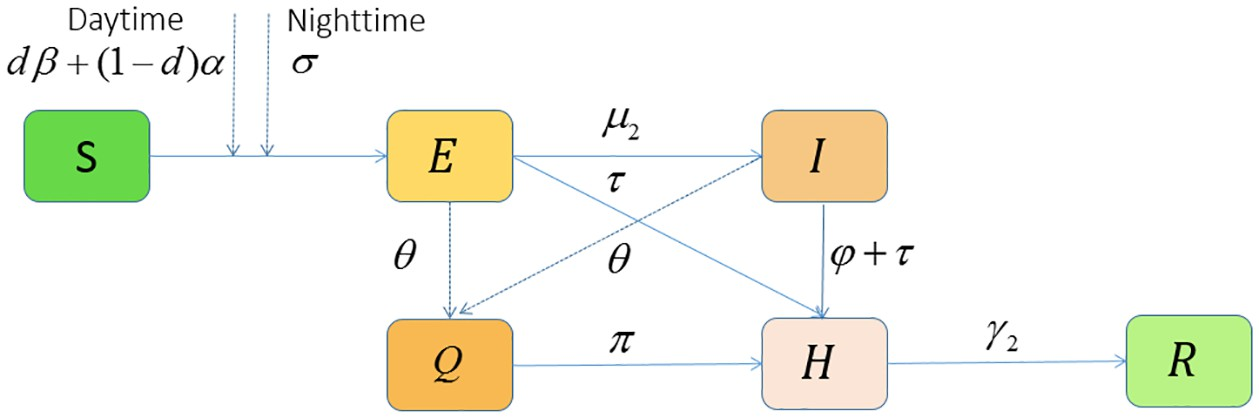
The flowchart of COVID-19 transmission with intervention measures.

NPIs should be taken as soon as a case is diagnosed. At this point, combined with the flowchart (Fig 2), we can establish the following model:

$$\begin{array}{c} \left\{ \begin{array}{cc} S_{k}^{a_{i}}\left(t\right) & =\left(1-f_{k}^{a_{i}}\left(t-1\right)\right)S_{k}^{a_{i}}\left(t-1\right),\\ E_{k}^{a_{i}}\left(t\right) & =f_{k}^{a_{i}}\left(t-1\right)\;S_{k}^{a_{i}}\left(t-1\right)+\left(1-\mu_{2}^{a_{i}}-\tau -\theta^{a_{i}}\right)E_{k}^{a_{i}}\left(t-1\right),\\ I_{k}^{a_{i}}\left(t\right) & =\mu_{2}^{a_{i}}E_{k}^{a_{i}}\left(t-1\right)+\left(1-\varphi^{a_{i}}-\tau -\theta^{a_{i}}\right)I_{k}^{a_{i}}\left(t-1\right),\\ Q_{k}^{a_{i}}\left(t\right) & =\theta^{a_{i}}E_{k}^{a_{i}}\left(t-1\right)+\theta^{a_{i}}I_{k}^{a_{i}}\left(t-1\right)+\left(1-\pi \right)Q_{k}^{a_{i}}\left(t-1\right)\\ H_{k}^{a_{i}}\left(t\right) & =\pi Q_{k}^{a_{i}}\left(t-1\right)+\tau E_{k}^{a_{i}}\left(t-1\right)+\left(\varphi^{a_{i}}+\tau \right)I_{k}^{a_{i}}\left(t-1\right)+\left(1-\gamma_{2}\right)H_{k}^{a_{i}}\left(t-1\right),\\ R_{k}^{a_{i}}\left(t\right) & =\gamma_{2}H_{k}^{a_{i}}\left(t-1\right)+R_{k}^{a_{i}}\left(t-1\right), \end{array}\right. \end{array}$$ (2)

Here, $Q_{k}^{a_{i}}\left(t\right)$ $\left(H_{k}^{a_{i}}\left(t\right)\right)$ represents the number of quarantined exposed and infectious individuals (nucleic acid test positive individuals) with household size-*k*, age group-*a*_*i*_ at time *t*. $\mu_{2}^{a_{i}}$ represents the rate of $E_{k}^{a_{i}}\to I_{k}^{a_{i}}$ without being traced or detected by nucleic acids test in society. *τ* represents the rate of $E_{k}^{a_{i}}\to H_{k}^{a_{i}}$ ($I_{k}^{a_{i}}\to H_{k}^{a_{i}}$) due to nucleic acid test in society. $\theta^{a_{i}}$ represents the rate of $E_{k}^{a_{i}}\to Q_{k}^{a_{i}}$ ($I_{k}^{a_{i}}\to Q_{k}^{a_{i}}$) due to tracing and quarantining. $\varphi^{a_{i}}$ represents the rate of $I_{k}^{a_{i}}\to H_{k}^{a_{i}}$ through active medical treatment. *π* represents the rate of $Q_{k}^{a_{i}}\to H_{k}^{a_{i}}$ through nucleic acid test in quarantine site. *γ*_2_ represents the recovery rate of $H_{k}^{a_{i}}$.

## Model analysis

Based on the established model, we can further calculate the basic reproduction number. It is the average number of secondary infected caused by an infectious individual in a completely susceptible population [18]. If it is greater than 1, the disease can spread and an outbreak can occur. If it is less than 1, the infection will decline in the population. According to [18–21], combined with the definition of the basic reproduction number, we can get that during daytime, the average number of secondary infected individuals caused by an infectious individual with household size-*k*, age group-*a*_*i*_ is:

$$\begin{array}{c} Y_{k}^{a_{i}}=\frac{5}{12}\left(1-d^{a_{i}}\right)\sum_{\iota }P\left(L_{k}^{a_{i}}=\iota \right)(\sum_{r}P\left(C_{k}^{a_{i}}=r|L_{k}^{a_{i}}=\iota \right)\sum_{l_{1},l_{2},\cdots ,l_{A}}\\ P(l_{1},l_{2},\cdots ,l_{A}|(C_{k}^{a_{i}}=r,L_{k}^{a_{i}}=\iota )P_{I}\left(t\right))+\frac{5}{12}d^{a_{i}}\sum_{j=1}^{A}(\frac{N^{a_{j}}X_{k}^{a_{i}}}{N})\lambda^{a_{j}}. \end{array}$$

Here, $P(l_{1},l_{2},\cdots ,l_{A}|C_{k}^{a_{i}}=r,L_{k}^{a_{i}}=\iota )$ represents the probability that of the *r* regular contacts, the number of $S^{a_{1}}$ is *l*_1_, the number of $S^{a_{2}}$ is *l*_2_ and the number of $S^{a_{A}}$ is *l*_*A*_. $P_{I}\left(t\right)=\left(l_{1}\lambda^{a_{1}}+l_{2}\lambda^{a_{2}}+\cdots +l_{A}\lambda^{a_{A}}\right)$ is the number of infected individuals. $\frac{N^{a_{j}}X_{k}^{a_{i}}}{N}$ represents the number of individuals with age group-*a*_*j*_ among the $X_{k}^{a_{i}}$ random contacts. At night, the number of secondary infected individuals of an infectious individual with household size-*k*, age group-*a*_*i*_ is:

$$Z_{k}^{a_{i}}=\frac{7}{12}\left(k-1\right)\sum_{{\bar{l}}_{1},{\bar{l}}_{2},\cdots ,{\bar{l}}_{A}}\left({\bar{l}}_{1}\lambda^{a_{1}}+{\bar{l}}_{2}\lambda^{a_{2}}+{\bar{l}}_{A}\lambda^{a_{A}}\right).$$

Here, ${\bar{l}}_{1}+{\bar{l}}_{2}+\cdots +{\bar{l}}_{A}=k-1$, represents that among the *k* − 1 family members, the number of $S^{a_{1}}$ is ${\bar{l}}_{1}$, the number of $S^{a_{2}}$ is ${\bar{l}}_{2}$, ⋯, and the number of $S^{a_{A}}$ is ${\tilde{l}}_{A}$.

Assume that *T* is the infection period and obeys exponential distribution, i.e. *T* ∼ *EXP*(*γ*_1_), *g*(*T*) = *γ*_1_*e*^−*γ*_1_*T*^. Therefore, the average infection period is:

$$\int_{0}^{\infty }Tg\left(T\right)dT=\frac{1}{\gamma_{1}}.$$

Thus, we get the basic reproduction number of system (1):

$$\begin{array}{c} R_{k,0}^{a_{i}}=\text{min}\{\sum_{\iota }P\left(L_{k}^{a_{i}}\left(t\right)=\iota \right)(\sum_{r}P\left(C_{k}^{a_{i}}\left(t\right)=r|L_{k}^{a_{i}}\left(t\right)=\iota \right)r)+\frac{5d^{a_{i}}\sum_{j=1}^{A}(\frac{N^{a_{j}}X_{k}^{a_{i}}}{N})\lambda^{a_{j}}}{12\gamma_{1}},\frac{Y_{k}^{a_{i}}}{\gamma_{1}}\}\\ +\text{min}\{k-1,\frac{Z_{k}^{a_{i}}}{\gamma_{1}}\}, \end{array}$$

which indicates the average number of secondary infected individuals caused by an infectious individual with household size-*k*, age group-*a*_*i*_. And,

(1) if

$$\frac{Y_{k}^{a_{i}}}{\gamma_{1}}\geq \sum_{\iota }P\left(L_{k}^{a_{i}}\left(t\right)=\iota \right)(\sum_{r}P\left(C_{k}^{a_{i}}\left(t\right)=r|L_{k}^{a_{i}}\left(t\right)=\iota \right)r)+\frac{5d^{a_{i}}\sum_{j=1}^{A}(\frac{N^{a_{j}}X_{k}^{a_{i}}}{N})\lambda^{a_{j}}}{12\gamma_{1}},$$

then,

$$\begin{array}{c} R_{k,0}^{a_{i}}=\sum_{\iota }P\left(L_{k}^{a_{i}}\left(t\right)=\iota \right)(\sum_{r}P\left(C_{k}^{a_{i}}\left(t\right)=r|L_{k}^{a_{i}}\left(t\right)=\iota \right)r)+\frac{5d^{a_{i}}\sum_{j=1}^{A}(\frac{N^{a_{j}}X_{k}^{a_{i}}}{N})\lambda^{a_{j}}}{12\gamma_{1}}\\ +\text{min}\{k-1,\frac{Z_{k}^{a_{i}}}{\gamma_{1}}\}, \end{array}$$

and,

$$\frac{dR_{k,0}^{a_{i}}}{dd^{a_{i}}}=\frac{5\sum_{j=1}^{A}(\frac{N^{a_{j}}X_{k}^{a_{i}}}{N})\lambda^{a_{j}}}{12\gamma_{1}}>0.$$

That is to say $R_{k,0}^{a_{i}}$ increases with the increase of $d^{a_{i}}$.

(2) if

$$\frac{Y_{k}^{a_{i}}}{\gamma_{1}}<\sum_{\iota }P\left(L_{k}^{a_{i}}\left(t\right)=\iota \right)(\sum_{r}P\left(C_{k}^{a_{i}}\left(t\right)=r|L_{k}^{a_{i}}\left(t\right)=\iota \right)r)+\frac{5d^{a_{i}}\sum_{j=1}^{A}(\frac{N^{a_{j}}X_{k}^{a_{i}}}{N})\lambda^{a_{j}}}{12\gamma_{1}},$$

then,

$$R_{k,0}^{a_{i}}=\frac{Y_{k}^{a_{i}}}{\gamma_{1}}+\text{min}\{k-1,\frac{Z_{k}^{a_{i}}}{\gamma_{1}}\},$$

and,

$$\begin{array}{c} \frac{dR_{k,0}^{a_{i}}}{dd^{a_{i}}}=-\frac{5}{12}\sum_{\iota }P\left(L_{k}^{a_{i}}=\iota \right)(\sum_{r}P\left(C_{k}^{a_{i}}=r|L_{k}^{a_{i}}=\iota \right)\sum_{l_{1},l_{2},\cdots ,l_{A}}P(l_{1},l_{2},\cdots ,l_{A}|(C_{k}^{a_{i}}=r,\\ \;\;L_{k}^{a_{i}}=\iota )P_{I}\left(t\right))+\frac{5}{12}\sum_{j=1}^{A}(\frac{N^{a_{j}}X_{k}^{a_{i}}}{N})\lambda^{a_{j}}. \end{array}$$

In this case, the increase or decrease of $R_{k,0}^{a_{i}}$ depends on the value of the expression on the right side of the equal sign.

Furthermore, we have:

$$R_{0}^{a_{i}}=\sum_{k=1}^{K}\frac{N_{k}^{a_{i}}}{N}R_{k,0}^{a_{i}},R_{k,0}=\sum_{i=1}^{A}\frac{N_{k}^{a_{i}}}{N}R_{k,0}^{a_{i}},R_{0}=\sum_{k=1}^{K}\sum_{i=1}^{A}\frac{N_{k}^{a_{i}}}{N}R_{k,0}^{a_{i}}.$$

Then,

$$\frac{dR_{0}^{a_{i}}}{dd^{a_{i}}}=\sum_{k=1}^{K}\frac{N_{k}^{a_{i}}}{N}\frac{dR_{k,0}^{a_{i}}}{dd^{a_{i}}},\frac{dR_{k,0}}{dd^{a_{i}}}=\sum_{i=1}^{A}\frac{N_{k}^{a_{i}}}{N}\frac{dR_{k,0}^{a_{i}}}{dd^{a_{i}}},\frac{dR_{0}}{dd^{a_{i}}}=\sum_{k=1}^{K}\sum_{i=1}^{A}\frac{N_{k}^{a_{i}}}{N}\frac{dR_{k,0}^{a_{i}}}{dd^{a_{i}}}.$$

According to the sign of the expression on the right side of the equal sign, we can judge the increase or decrease of each basic reproduction number with respect to the proportion of random contacts.

For the system (2) with intervention measures, the expression of the control reproduction number can be obtained by a similar method:

$$\begin{array}{c} R_{k,c}^{a_{i}}=\text{min}\{\sum_{\iota }P\left(L_{k}^{a_{i}}\left(t\right)=\iota \right)(\sum_{r}P\left(C_{k}^{a_{i}}\left(t\right)=r|L_{k}^{a_{i}}\left(t\right)=\iota \right)r)+\frac{5d^{a_{i}}\sum_{j=1}^{A}(\frac{N^{a_{j}}X_{k}^{a_{i}}}{N})\lambda^{a_{j}}}{12(\varphi^{a_{i}}+\tau +\theta^{a_{i}})},\frac{Y_{k}^{a_{i}}}{\varphi^{a_{i}}+\tau +\theta^{a_{i}}}\}\\ +\text{min}\{k-1,\frac{Z_{k}^{a_{i}}}{\varphi^{a_{i}}+\tau +\theta^{a_{i}}}\}. \end{array}$$

Then,

$$R_{c}^{a_{i}}=\sum_{k=1}^{K}\frac{N_{k}^{a_{i}}}{N}R_{k,c}^{a_{i}},R_{k,c}=\sum_{i=1}^{A}\frac{N_{k}^{a_{i}}}{N}R_{k,c}^{a_{i}},R_{c}=\sum_{k=1}^{K}\sum_{i=1}^{A}\frac{N_{k}^{a_{i}}}{N}R_{k,c}^{a_{i}}.$$

Similarity, we can judge the increase or decrease of each control reproduction number with respect to the proportion of random contacts.

## Model application

We conducted our study based on the outbreak of COVID-19 epidemic in Yangzhou City in July 2021. The epidemic began to spread freely among population on July 21st, 2021, and the free transmission stage did not end until two confirmed cases were reported on July 28th. Since then, the epidemic spread rapidly. Meanwhile, the Yangzhou municipal government took a series of NPIs, including tracing and quarantining close contacts, isolation of confirmed cases, mass nucleic acid testing, and so on. Under strict control measures, the last confirmed case was reported on August 26th. The outbreak lasted more than 40 days and caused a total of 570 infected individuals. This section we will estimate the parameters of the model, and evaluate the duration of free transmission stage, age heterogeneity, different contact patterns and summer vacation on the spread of COVID-19.

### Data analysis

**Fig 3:**
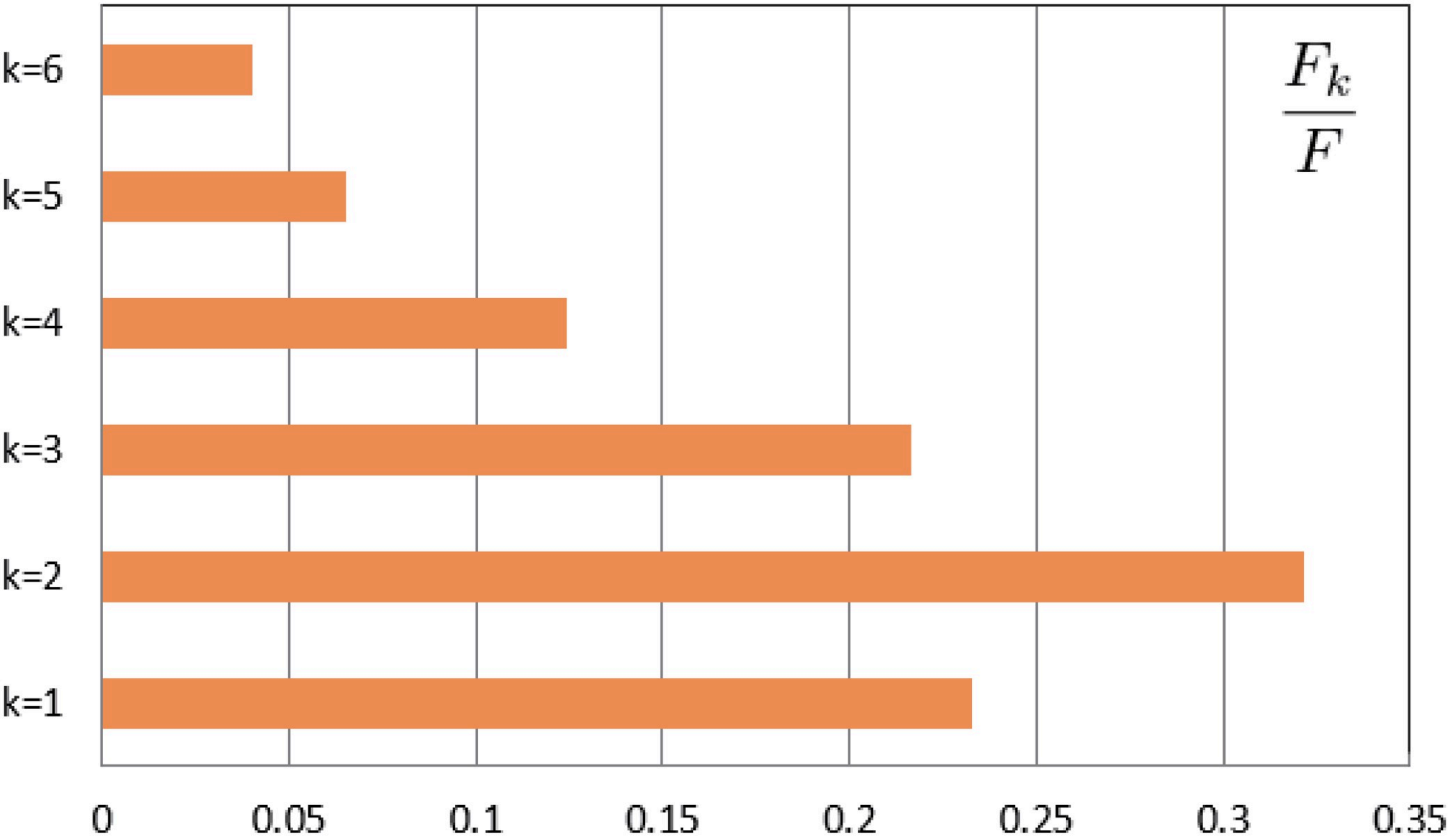
The distribution of household size in Yangzhou City.

**Fig 4:**
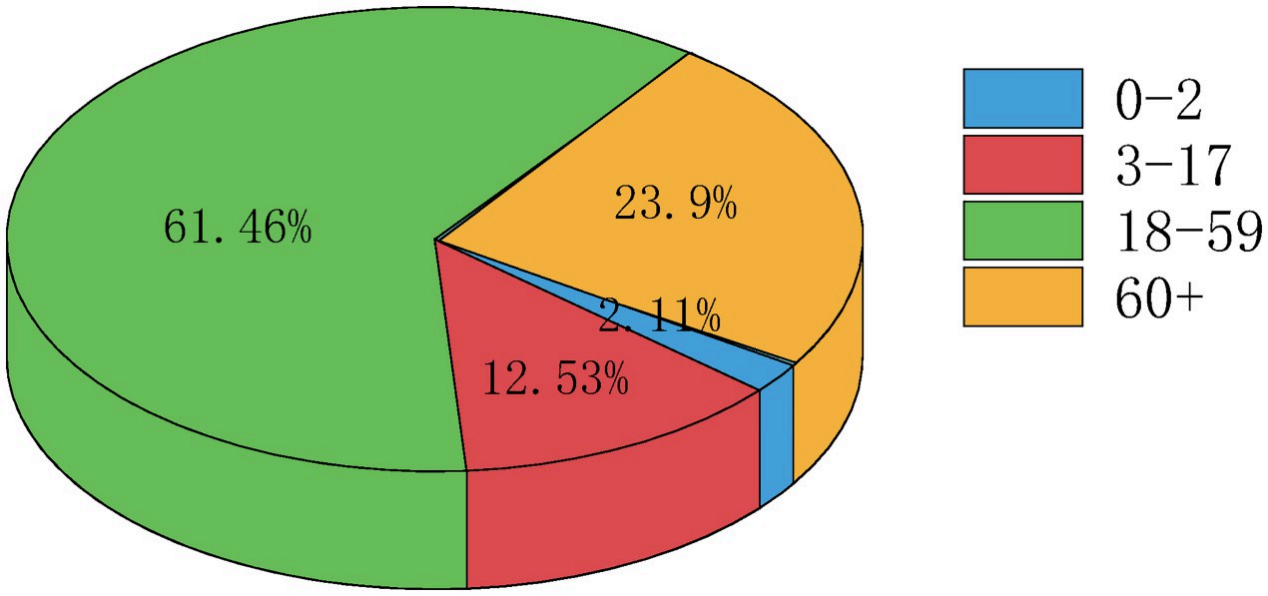
Age distribution of population in Yangzhou City.

The population and households in Yangzhou City are divided as follows:

1. According to the China Statistical Yearbook (2021) [22], all households in Jiangsu Province are divided into ten categories according to different sizes, which are households with size of one, two, three, four, five, six, seven, eight, nine, ten and above, and then obtain the proportion of different household sizes. It is assumed that the household structure of Yangzhou City is the same as that of Jiangsu Province, and households with size greater than six fall into the category of six. In addition, the collective household population is allocated to each kind of households according to the proportion of the population contained in each kind of households. Then, we can get the number of households with size of *k*: *F*_*k*_ (*k* = 1, 2, 3, 4, 5, 6). Fig 3 gives the household distribution of Yangzhou City. In addition, all the population of Yangzhou City is also divided into six categories according to the household size, and *N*_*k*_ (*k* = 1, 2, 3, 4, 5, 6) represents the number of individuals with household size-*k*.
2. According to the Statistical Yearbook of Jiangsu Province (2021) [23] and Yangzhou Statistical Yearbook (2021), the total population we studied of Yangzhou City is 4.561 million. And the population of Yangzhou is divided into four categories by age: [0, 2], [3, 17], [18, 59], 60+, and $N^{a_{i}}$ represents the number of individuals with age group-*a*_*i*_. Age distribution of population in Yangzhou City can be found in Fig 4.
3. For each household, there is no more detailed data on the age composition. According to the data provided by Statistical Yearbook of Jiangsu Province (2021) [23], Yangzhou Statistical Yearbook (2021) [24], China Population Census Yearbook (2020) [25], and make some assumptions, we can get the age composition of the family members of Yangzhou City. According to the actual situation, we consider that only individuals in age group-*a*_3_ or age group-*a*_4_ can form a household with size-1. Furthermore, according to China Population Census Yearbook (2020) [25], we get the proportion of individuals in age group-*a*_4_ living alone in Jiangsu Province, the proportion of two individuals in age group *a*_4_ living together, as well as the proportion of individuals in age group *a*_4_ who live together with minors (age group *a*_1_ or *a*_2_). Assuming that the three proportions of Yangzhou City are the same as those of Jiangsu Province, then we can get $F_{1}^{a_{3}}=N_{1}^{a_{3}}=259,588,F_{1}^{a_{4}}=N_{1}^{a_{4}}=159,441$, $F_{2}^{a_{4}a_{4}}=192,764$, $F_{2}^{a_{1}a_{4}}=733,F_{2}^{a_{2}a_{4}}=4396$ in Yangzhou City. Here $F_{2}^{a_{i}a_{j}}$ represents the number of families of household size 2 consisting of two individuals with age group-*a*_*i*_ and age group-*a*_*j*_. Similarly, we can get the meaning of the other terms.
  Based on the divorce and bereavement rates in Jiangsu Province, it is calculated that $F_{2}^{a_{13}}=7,046,F_{2}^{a_{23}}=42,277$ in Yangzhou City. Assuming that $F_{2}^{a_{4}a_{4}}$ is about ten times of $F_{2}^{a_{3}a_{4}}$, we have $F_{2}^{a_{3}a_{4}}=1,927$. Then we have $F_{2}^{a_{3}a_{3}}=361,174$. For the age composition of households with size greater than 2, we first allocate the remaining individuals in each age group according to the proportion of the population included in household of each size. Then, based on the results of the WeChat Questionnaire Star Mini Program, we obtained the age composition of household with size larger than 2. The specific results are shown in Table 1.

**Table 1:** The detailed structure and number of households.

*F*_1_	$F_{1}^{a_{3}}$	$F_{1}^{a_{4}}$			
410, 029	259, 588	15,0441			
*F*_2_	$F_{2}^{a_{1}a_{3}}$	$F_{2}^{a_{1}a_{4}}$	$F_{2}^{a_{2}a_{3}}$	$F_{2}^{a_{2}a_{4}}$	$F_{2}^{a_{3}a_{3}}$
566, 405	7, 046	733	42, 277	4, 396	309, 551
	$F_{2}^{a_{3}a_{4}}$	$F_{2}^{a_{4}a_{4}}$			
	9, 638	192, 764			
*F*_3_	$F_{3}^{a_{1}a_{3}a_{3}}$	$F_{3}^{a_{1}a_{4}a_{4}}$	$F_{3}^{a_{2}a_{3}a_{3}}$	$F_{3}^{a_{2}a_{4}a_{4}}$	$F_{3}^{a_{1}a_{2}a_{3}}$
382, 062	27, 796	1, 240	187, 244	7, 440	4, 633
	$F_{3}^{a_{3}a_{3}a_{3}}$	$F_{3}^{a_{3}a_{3}a_{4}}$	$F_{3}^{a_{3}a_{4}a_{4}}$		
	57,698	4, 633	91, 378		
*F*_4_	$F_{4}^{a_{1}a_{2}a_{3}a_{3}}$	$F_{4}^{a_{1}a_{3}a_{3}a_{3}}$	$F_{4}^{a_{1}a_{3}a_{4}a_{4}}$	$F_{4}^{a_{2}a_{2}a_{3}a_{3}}$	$F_{4}^{a_{2}a_{3}a_{3}a_{3}}$
218, 549	15, 409	5, 135	5, 135	44, 068	22, 034
	$F_{4}^{a_{2}a_{3}a_{3}a_{4}}$	$F_{4}^{a_{2}a_{3}a_{4}a_{4}}$	$F_{4}^{a_{3}a_{3}a_{3}a_{4}}$	$F_{4}^{a_{3}a_{3}a_{4}a_{4}}$	$F_{4}^{a_{3}a_{3}a_{3}a_{3}}$
	22, 034	4, 406	23, 010	46, 018	31, 300
*F*_5_	$F_{5}^{a_{1}a_{3}a_{3}a_{3}a_{3}}$	$F_{5}^{a_{1}a_{2}a_{3}a_{3}a_{4}}$	$F_{5}^{a_{2}a_{3}a_{3}a_{3}a_{3}}$	$F_{5}^{a_{2}a_{2}a_{3}a_{3}a_{3}}$	$F_{5}^{a_{2}a_{3}a_{3}a_{3}a_{4}}$
115, 047	12, 673	4, 224	20, 712	18, 123	2, 589
	$F_{5}^{a_{2}a_{2}a_{3}a_{3}a_{4}}$	$F_{5}^{a_{2}a_{3}a_{3}a_{4}a_{4}}$	$F_{5}^{a_{3}a_{3}a_{3}a_{4}a_{4}}$	$F_{5}^{a_{3}a_{3}a_{3}a_{3}a_{4}}$	$F^{a_{3}a_{3}a_{3}a_{3}a_{3}}$
	7, 767	20, 726	7, 787	7, 790	4, 869
	$F_{5}^{a_{3}a_{3}a_{4}a_{4}a_{4}}$				
	7, 787				
*F*_6_	$F_{6}^{a_{1}a_{3}a_{3}a_{3}a_{3}a_{3}}$	$F_{6}^{a_{1}a_{1}a_{3}a_{3}a_{4}a_{4}}$	$F_{6}^{a_{1}a_{2}a_{3}a_{3}a_{4}a_{4}}$	$F_{6}^{a_{2}a_{2}a_{3}a_{3}a_{3}a_{4}}$	$F_{6}^{a_{2}a_{2}a_{3}a_{3}a_{4}a_{4}}$
70, 424	3, 103	3, 103	3, 103	5, 251	15, 768
	$F_{6}^{a_{2}a_{3}a_{3}a_{3}a_{4}a_{4}}$	$F_{6}^{a_{2}a_{3}a_{3}a_{4}a_{4}a_{4}}$	$F_{6}^{a_{2}a_{2}a_{3}a_{3}a_{3}a_{3}}$	$F_{6}^{a_{3}a_{3}a_{3}a_{3}a_{3}a_{3}}$	
	5, 256	5, 257	8, 912	20, 671	

We used the WeChat Questionnaire Star Mini program to investigate the age composition of household members. Excluding some useless data, data from 186 households was obtained and applied to Yangzhou City. These can reflect the common age composition of household members. Also, to ensure the completeness of the grouping, we may add some structures that are not in the Questionnaire.

### Infection probability

In the following, we will give the detailed expression of the infection probability based on the above data.

**Fig 5:**
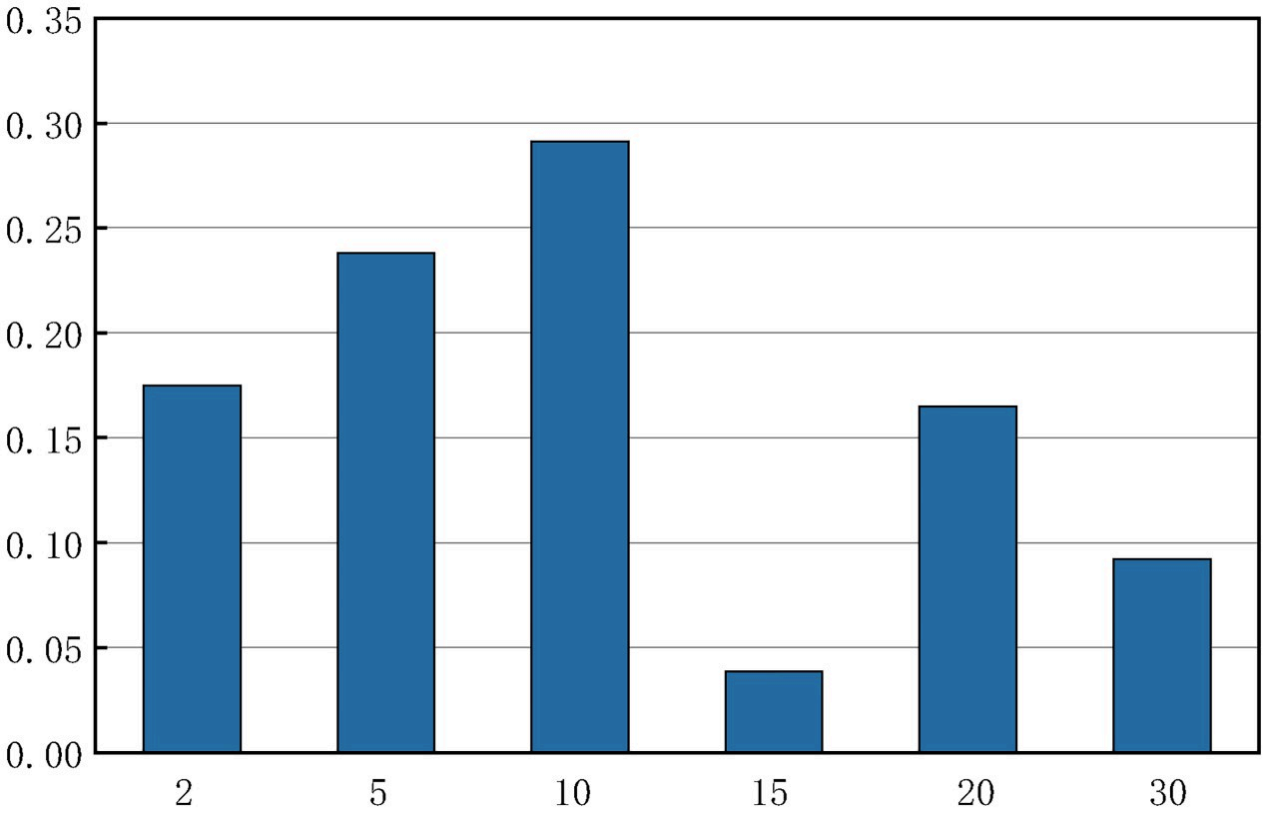
Distribution of the number of regular contacts in the workplace.

The outbreak of COVID-19 in Yangzhou City coincides with the summer vacation, and the article does not consider the spread within school. Then, let’s make the following assumptions:

1. During daytime, minors (age group-*a*_1_ or *a*_2_) have regular contacts with at least one adult (age group-*a*_3_ or *a*_4_).
2. If households are made up of minors and one or two individuals in age group-*a*_3_, based on the results of the Questionnaire, households with proportion *h* (*h* = 0.45) have only one individual in age group-*a*_3_ who take care of minors at home full-time. The individuals with age group-*a*_3_ in the remaining 1 − *h* households are all employed, and assume that minors in those households with proportion *c* (assume *c* = 0.5) are taken care of by a individual with household size-1, age group-*a*_4_ and 1 − *c* by two individuals with household size-2, age group-*a*_4_ who come from the same family. During daytime, the employed individuals with age group-*a*_3_ have regular contacts with employed individuals with age group-*a*_3_ in workplace, and the number of regular contacts of each individual is an independent and identically distributed random variable. While conducting the survey on the age composition of household members, we also added an option on the number of regular contacts in workplace, and got 206 valid data. The data was statistically processed to obtain the distribution of the number of regular contacts in the workplace, as shown in Fig 5.
3. If households are composed of minors and three or more individuals in age group-*a*_3_, then it is assumed that only one individual with age group-*a*_3_ in those households is unemployed and take care of minors at home full-time during daytime.
4. If individuals in age group-*a*_3_ and age group-*a*_4_ are in the same household, then the place of regular contacts of individuals with age group-*a*_3_ is the workplace during daytime. Individuals with age-group *a*_4_ who do not have regular contact with minors shall have regular contact with the same type of individuals at activity place with proportion of *q*_1_. If a household contains minors and three or more individuals with age group-*a*_4_, a proportion of *q*_2_ households have only one individual with age group *a*_4_ making regular contact at the activity place.

Based on the previous assumptions and the data collected, we can obtain the infection probability with regular contacts during daytime:

when *i* = 1:

$$\begin{array}{c} \left\{ \begin{array}{cc} \alpha_{2}^{a_{1}}\left(t\right) & =1-\frac{hF_{2}^{a_{1}a_{3}}}{N_{2}^{a_{1}}}\left(1-\lambda^{a_{1}}{\bar{I}}_{2}^{a_{3}}\left(t\right)\right)-\frac{F_{2}^{a_{1}a_{4}}}{N_{2}^{a_{1}}}\left(1-\lambda^{a_{1}}{\bar{I}}_{2}^{a_{4}}\left(t\right)\right)-\frac{c\left(1-h\right)F_{2}^{a_{1}a_{3}}}{N_{2}^{a_{1}}}\left(1-\lambda^{a_{1}}{\bar{I}}_{1}^{a_{4}}\left(t\right)\right)\\ & -\frac{\left(1-c\right)\left(1-h\right)F_{2}^{a_{1}a_{3}}}{N_{2}^{a_{1}}}{\left(1-\lambda^{a_{1}}{\bar{I}}_{2}^{a_{4}}\left(t\right)\right)}^{2},\\ \alpha_{3}^{a_{1}}\left(t\right) & =1-\frac{hF_{3}^{a_{1}a_{3}a_{3}}}{N_{3}^{a_{1}}}\left(1-\lambda^{a_{1}}{\bar{I}}_{3}^{a_{3}}\left(t\right)\right)-\frac{c\left(1-h\right)F_{3}^{a_{1}a_{3}a_{3}}}{N_{3}^{a_{1}}}\left(1-\lambda^{a_{1}}{\bar{I}}_{1}^{a_{4}}\left(t\right)\right)\\ & -\frac{\left(1-c\right)\left(1-h\right)F_{3}^{a_{1}a_{3}a_{3}}}{N_{3}^{a_{1}}}{\left(1-\lambda^{a_{1}}{\bar{I}}_{2}^{a_{4}}\left(t\right)\right)}^{2}-\frac{F_{3}^{a_{1}a_{4}a_{4}}}{N_{3}^{a_{1}}}{\left(1-\lambda^{a_{1}}{\bar{I}}_{3}^{a_{4}}\left(t\right)\right)}^{2}\\ & -\frac{hF_{3}^{a_{1}a_{2}a_{3}}}{N_{3}^{a_{1}}}\left(1-\lambda^{a_{1}}{\bar{I}}_{3}^{a_{2}}\left(t\right)\right)\left(1-\lambda^{a_{1}}{\bar{I}}_{3}^{a_{3}}\left(t\right)\right)-\frac{c\left(1-h\right)F_{3}^{a_{1}a_{2}a_{3}}}{N_{3}^{a_{1}}}\left(1-\lambda^{a_{1}}{\bar{I}}_{3}^{a_{2}}\left(t\right)\right)\\ & \left(1-\lambda^{a_{1}}{\bar{I}}_{1}^{a_{4}}\left(t\right)\right)-\frac{\left(1-c\right)\left(1-h\right)F_{3}^{a_{1}a_{2}a_{3}}}{N_{3}^{a_{1}}}\left(1-\lambda^{a_{1}}{\bar{I}}_{3}^{a_{2}}\left(t\right)\right){\left(1-\lambda^{a_{1}}{\bar{I}}_{2}^{a_{4}}\left(t\right)\right)}^{2},\\ \alpha_{4}^{a_{1}}\left(t\right) & =1-\frac{F_{4}^{a_{1}a_{3}a_{3}a_{3}}}{N_{4}^{a_{1}}}\left(1-\lambda^{a_{1}}{\bar{I}}_{4}^{a_{3}}\left(t\right)\right)-\frac{hF_{4}^{a_{1}a_{2}a_{3}a_{3}}}{N_{4}^{a_{1}}}\left(1-\lambda^{a_{1}}{\bar{I}}_{4}^{a_{2}}\left(t\right)\right)\left(1-\lambda^{a_{1}}{\bar{I}}_{4}^{a_{3}}\left(t\right)\right)\\ & -\frac{c\left(1-h\right)F_{4}^{a_{1}a_{2}a_{3}a_{3}}}{N_{4}^{a_{1}}}\left(1-\lambda^{a_{1}}{\bar{I}}_{4}^{a_{2}}\left(t\right)\right)\left(1-\lambda^{a_{1}}{\bar{I}}_{1}^{a_{4}}\left(t\right)\right)-\frac{\left(1-c\right)\left(1-h\right)F_{4}^{a_{1}a_{2}a_{3}a_{3}}}{N_{4}^{a_{1}}}\\ & \left(1-\lambda^{a_{1}}{\bar{I}}_{4}^{a_{2}}\left(t\right)\right){\left(1-\lambda^{a_{1}}{\bar{I}}_{2}^{a_{4}}\left(t\right)\right)}^{2}-\frac{F_{4}^{a_{1}a_{3}a_{4}a_{4}}}{N_{4}^{a_{1}}}{\left(1-\lambda^{a_{1}}{\bar{I}}_{4}^{a_{4}}\left(t\right)\right)}^{2},\\ \alpha_{5}^{a_{1}}\left(t\right) & =1-\frac{F_{5}^{a_{1}a_{3}a_{3}a_{3}a_{3}}}{N_{5}^{a_{1}}}\left(1-\lambda^{a_{1}}{\bar{I}}_{5}^{a_{3}}\left(t\right)\right)-\frac{F_{5}^{a_{1}a_{2}a_{3}a_{3}a_{4}}}{N_{5}^{a_{1}}}\left(1-\lambda^{a_{1}}{\bar{I}}_{5}^{a_{2}}\left(t\right)\right)\left(1-\lambda^{a_{1}}{\bar{I}}_{5}^{a_{4}}\left(t\right)\right),\\ \alpha_{6}^{a_{1}}\left(t\right) & =1-\frac{F_{6}^{a_{1}a_{3}a_{3}a_{3}a_{3}a_{3}}}{N_{6}^{a_{1}}}\left(1-\lambda^{a_{1}}{\bar{I}}_{6}^{a_{3}}\left(t\right)\right)-\frac{2F_{6}^{a_{1}a_{1}a_{3}a_{3}a_{4}a_{4}}}{N_{6}^{a_{1}}}\left(1-\lambda^{a_{1}}{\bar{I}}_{6}^{a_{1}}\left(t\right)\right){\left(1-\lambda^{a_{1}}{\bar{I}}_{6}^{a_{4}}\left(t\right)\right)}^{2}\\ & -\frac{F_{6}^{a_{1}a_{2}a_{3}a_{3}a_{4}a_{4}}}{N_{6}^{a_{1}}}\left(1-\lambda^{a_{1}}{\bar{I}}_{6}^{a_{2}}\left(t\right)\right){\left(1-\lambda^{a_{1}}{\bar{I}}_{6}^{a_{4}}\left(t\right)\right)}^{2}. \end{array}\right. \end{array}$$

Here, ${\bar{I}}_{k}^{a_{i}}\left(t\right)=\frac{I_{k}^{a_{i}}\left(t\right)}{N_{k}^{a_{i}}}$ represents the proportion of infectious individuals with household size-k, age group-*a*_*i*_. The second term on the right of the first formula represents the probability of a susceptible individual with household size-2, age group-*a*_1_ not being infected who has regular contacts with an infectious individual with age group-*a*_3_ in the same household. The third term indicates that the probability of the susceptible individual not being infected who has regular contacts with an infectious individual with age group-*a*_4_ in the same household. The forth term indicates that the probability of the susceptible individual not being infected who has regular contacts with an infectious individual with household size-1, age group-*a*_4_. These three terms can be derived as follows:

$$P\left(L_{2}^{a_{1}}\left(t\right)=HO\right)P\left(C_{2}^{a_{1}}\left(t\right)=1|L_{2}^{a_{1}}\left(t\right)=HO\right)P\left(\text{Uninfected}\;\text{during}\;\text{daytime}|C_{2}^{a_{1}}\left(t\right)=1,L_{2}^{a_{1}}\left(t\right)=\text{HO}\right)$$

$$=1\times \frac{\bar{W}}{N_{2}^{a_{1}}}\times (\frac{hF_{2}^{a_{1}a_{3}}}{\bar{W}}\left(1-\lambda^{a_{1}}{\bar{I}}_{2}^{a_{3}}\left(t\right)\right)+\frac{F_{2}^{a_{1}a_{4}}}{\bar{W}}\left(1-\lambda^{a_{1}}{\bar{I}}_{2}^{a_{4}}\left(t\right)\right)+\frac{c\left(1-h\right)F_{2}^{a_{1}a_{3}}}{\bar{W}}\left(1-\lambda^{a_{1}}{\bar{I}}_{1}^{a_{4}}\left(t\right)\right))$$

$$=\frac{hF_{2}^{a_{1}a_{3}}}{N_{2}^{a_{1}}}\left(1-\lambda^{a_{1}}{\bar{I}}_{2}^{a_{3}}\left(t\right)\right)+\frac{F_{2}^{a_{1}a_{4}}}{N_{2}^{a_{1}}}\left(1-\lambda^{a_{1}}{\bar{I}}_{2}^{a_{4}}\left(t\right)+\frac{c\left(1-h\right)F_{2}^{a_{1}a_{3}}}{N_{2}^{a_{1}}}\left(1-\lambda^{a_{1}}{\bar{I}}_{1}^{a_{4}}\left(t\right)\right)\right).\;\;\;\;$$

Here, $\bar{W}=hF_{2}^{a_{1}a_{3}}+F_{2}^{a_{1}a_{4}}+c\left(1-h\right)F_{2}^{a_{1}a_{3}}$. The fifth item means the probability of not being infected of the individual who has regular contacts with two infectious individuals with household size-2, age group-*a*_4_ come from the same family. The first expression, on the whole, represents the average probability of being infected of a susceptible individual with household size-2, age group-*a*_1_. Similarly, the meaning of other expressions can be obtained.

As for the other age groups, the expressions of infection probability with regular contacts during daytime are shown in Supporting information (S1 File).

Regular contacts at night mainly depend on the household size, so we classify the probability of being infected due to regular contacts according to the household size.

1. When *k* = 1:
  $$\sigma_{1}^{a_{3}}=\sigma_{1}^{a_{4}}=0.$$
2. When *k* = 2:
  $$\sigma_{2}^{a_{i}}\left(t\right)=1-\sum_{j}\frac{F_{2}^{a_{i}a_{j}}}{N_{2}^{a_{i}}}\left(1-\lambda^{a_{i}}{\bar{I}}_{2}^{a_{j}}\left(t\right)\right),i\in \left\{1,2,3,4\right\}.$$
3. When *k* = 3:
  $$\sigma_{3}^{a_{i}}\left(t\right)=1-\sum_{j_{1},j_{2}}\frac{F_{3}^{a_{i}a_{j_{1}}a_{j_{2}}}}{N_{3}^{a_{i}}}\left(1-\lambda^{a_{i}}{\bar{I}}_{3}^{a_{j_{1}}}\left(t\right)\right)\left(1-\lambda^{a_{i}}{\bar{I}}_{3}^{a_{j_{2}}}\left(t\right)\right),i\in \left\{1,2,3,4\right\}.$$
4. When *k* = 4:
  $$\begin{array}{c} \sigma_{4}^{a_{i}}\left(t\right)=1-\sum_{j_{1},j_{2},j_{3}}\frac{F_{3}^{a_{i}a_{j_{1}}a_{j_{2}}a_{j_{3}}}}{N_{4}^{a_{i}}}\left(1-\lambda^{a_{i}}{\bar{I}}_{4}^{a_{j_{1}}}\left(t\right)\right)\left(1-\lambda^{a_{i}}{\bar{I}}_{4}^{a_{j_{2}}}\left(t\right)\right)\left(1-\lambda^{a_{i}}{\bar{I}}_{4}^{a_{j_{3}}}\left(t\right)\right),\\ i\in \{1,2,3,4\}. \end{array}$$
5. When *k* = 5:
  $$\begin{array}{c} \sigma_{5}^{a_{i}}\left(t\right)=1-\sum_{j_{1},j_{2},j_{3},j_{4}}\frac{F_{5}^{a_{i}a_{j_{1}}a_{j_{2}}a_{j_{3}}a_{j_{4}}}}{N_{5}^{a_{i}}}\left(1-\lambda^{a_{i}}{\bar{I}}_{5}^{a_{j_{1}}}\left(t\right)\right)\left(1-\lambda^{a_{i}}{\bar{I}}_{5}^{a_{j_{2}}}\left(t\right)\right)\left(1-\lambda^{a_{i}}{\bar{I}}_{5}^{a_{j_{3}}}\left(t\right)\right)\\ (1-\lambda^{a_{i}}{\bar{I}}_{5}^{a_{j_{4}}}\left(t\right)),i\in \left\{1,2,3,4\right\}. \end{array}$$
6. When *k* = 6:
  $$\begin{array}{c} \sigma_{6}^{a_{i}}\left(t\right)=1-\sum_{j_{1},j_{2},j_{3},j_{4},j_{5}}\frac{F_{6}^{a_{i}a_{j_{1}}a_{j_{2}}a_{j_{3}}a_{j_{4}}a_{j_{5}}}}{N_{6}^{a_{i}}}\left(1-\lambda^{a_{i}}{\bar{I}}_{6}^{a_{j_{1}}}\left(t\right)\right)\left(1-\lambda^{a_{i}}{\bar{I}}_{6}^{a_{j_{2}}}\left(t\right)\right)\left(1-\lambda^{a_{i}}{\bar{I}}_{6}^{a_{j_{3}}}\left(t\right)\right)\\ \left(1-\lambda^{a_{i}}{\bar{I}}_{6}^{a_{j_{4}}}\left(t\right)\right)\left(1-\lambda^{a_{i}}{\bar{I}}_{6}^{a_{j_{5}}}\left(t\right)\right),i\in \left\{1,2,3,4\right\}. \end{array}$$

**Fig 6:**
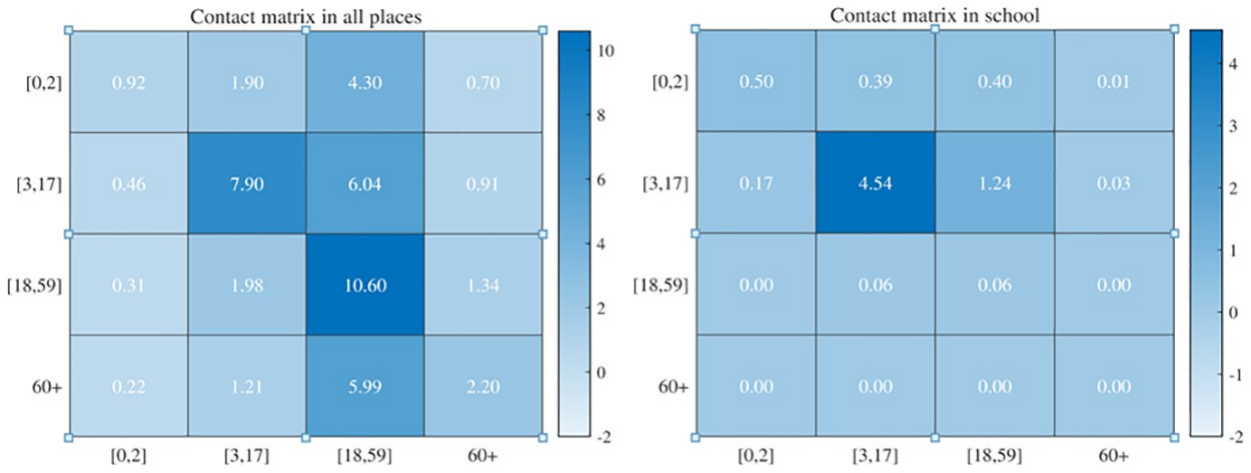
The figures of contact matrix. The left figure represents the contacts made at all places. The right figure represents the contacts made at school.

It is assumed that the number of random contacts of individuals during daytime depends only on age. Prem K et al. [26] given the number of contacts in the 16 age groups. According to the principle of total number of contacts in different grouping methods are equal, we obtained the number of contacts under the current grouping (Fig 6). Each row of the contact matrix is added together to obtain the number of total contacts at all places for each age group. Then subtract the number of contacts at school from the number of contacts at all places, subtract the average number of family members, and subtract the number of regular contacts during the daytime excluding the average number of family members, and we obtain an approximation of random contacts of each age group: $X^{a_{1}}=3.83,X^{a_{2}}=6.65,X^{a_{3}}=4.87,X^{a_{4}}=4.33$.

Thus, for each individual with household size-*k*, age group-*a*_*i*_, the probability of being infected by random contacts at time *t* is:

$$\beta^{a_{i}}\left(t\right)=1-{\left(1-\lambda^{a_{i}}\frac{I(t)}{N}\right)}^{X^{a_{i}}},i=1,2,3,4.$$

### Numerical simulations

**Fig 7:**
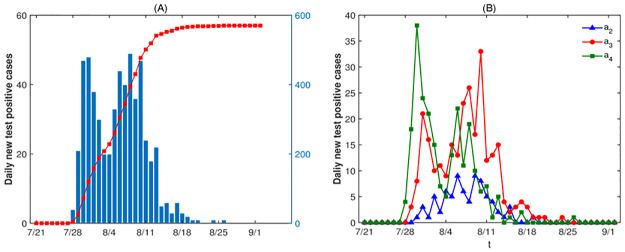
(A) The number of daily new test positive cases and cumulative test positive cases for all age groups. (B) The number of daily new test positive cases for age group-*a*_*i*_, *i* = 2, 3, 4.

After the emergence of confirmed cases in July 28th, the Yangzhou municipal government immediately took a series of NPIs. Then control measures were implemented in the main urban area on July 31st, and closed management was carried out in the communities with confirmed cases [27]. Due to the grim situation of the epidemic, closed management was implemented in all communities on August 3rd [28]. Under strict control measures, the last confirmed case was reported on August 26th, and on September 3rd, the closed control area no longer implemented access card management, and enterprises began to resume work and production in an orderly manner. A total of 570 people were infected in this epidemic (daily new test positive cases and cumulative test positive cases of Yangzhou City are shown in Fig 7). Therefore, we divide the research time into four stages: stage 1 is from July 21st to July 27th, which corresponds to the free transmission stage of the disease, and can be described by system (1); stage 2 is from July 28th to August 2ed, which is the initial implementation stage of NPIs, and can be described by model (2); stage 3 is from August 3rd to August 9th corresponds to a further strengthening phase of NPIs; stage 4 is from August 10th to September 2ed when the intensity of NPIs are the strongest. In stage 3 and stage 4, most enterprises and factories have stopped production. Each family is limited to one person per day with a pass to go out to purchase daily necessities. We assume that there is neither contact in workplace nor contact in activity place in these two stage, and individuals only have regular contacts with family members. Meanwhile the number of random contacts is also greatly reduced, and we assume that the number of random contacts is the same for all age groups. And we set $X^{a_{1}}=X^{a_{2}}=X^{a_{3}}=X^{a_{4}}=1$ in stage 3 and stage 4. Stage 3 and stage 4 can be represented by model (2), but $f_{k}^{a_{i}}\left(t\right)$ becomes:

$$f_{k}^{a_{i}}\left(t\right)=\{\begin{array}{c} d^{a_{i}}\beta_{k}^{a_{i}}\left(t\right)+\left(1-d^{a_{i}}\right)\sigma_{k}^{a_{i}}\left(t\right),\text{daytime},\\ \sigma_{k}^{a_{i}}\left(t\right),\text{nighttime}. \end{array}$$

Since there are only 5 reported cases of age group-*a*_1_, and there is no significant difference in susceptibility between individuals with aged group-*a*_1_ and age group-*a*_2_, so in the later study, the two age groups were combined into one age group, represented by *a*_2_. The number of random contacts of the new age group is taken as the weighted average of the privious two age groups, that is $X^{a_{2}}=6.24$ in stage 1 and stage 2. In stage 3 and stage 4, we also take $X^{a_{2}}=1$. In addition, based on the actual situation we set $d^{a_{2}}=d^{a_{3}}=d^{a_{4}}=0.4$ in stage 1 and stage 2, $d^{a_{2}}=d^{a_{3}}=d^{a_{4}}=0.05$ in stage 3 and stage 4. In addition, we assume $q=q_{1}=q_{2}=\frac{2}{3}$ and the number of regular contacts of the elderly (age group-*a*_4_) in the activity place is 7.

Assume that ${ML}^{a_{i}}\left(t\right)$ represents cumulative test positive cases of age group-*a*_*i*_ predicted by the model, and can be expressed as follows:

$${ML}^{a_{i}}\left(t\right)=\sum_{k=1}^{6}\left(\pi Q_{k}^{a_{i}}\left(t-1\right)+\tau E_{k}^{a_{i}}\left(t-1\right)+\left(\varphi^{a_{i}}+\tau \right)I_{k}^{a_{i}}\left(t-1\right)+H_{k}^{a_{i}}\left(t-1\right)\right),i=2,3,4.$$

**Fig 8:**
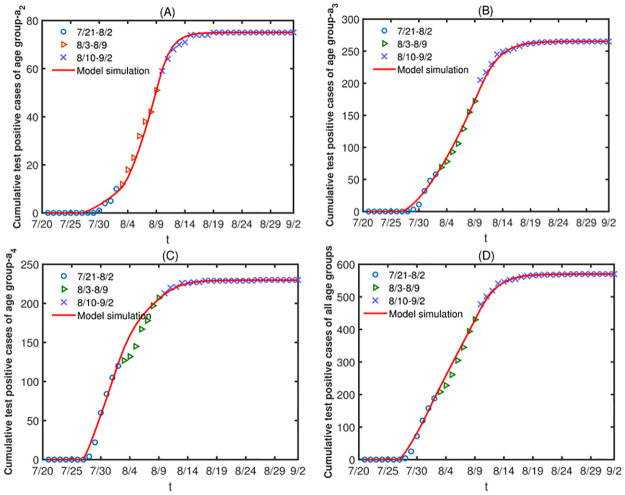
(A) The fitting results of age group-*a*_2_. (B) The fitting results of age group-*a*_3_. (C) The fitting results of age group-*a*_4_. (D) The results of the total population.

${\hat{ML}}^{a_{i}}\left(t\right)$ represents the reported cumulative test positive cases of age group-*a*_*i*_ per day. Then we use the least square (LS) method to find the parameter value to minimize the objective function [29, 30]:

$$J\left(\Phi \right)=\sum_{i=2}^{4}(\sum_{t=1}^{n}\frac{{\hat{ML}}^{a_{i}}\left(n\right)}{\sum_{i=2}^{4}{\hat{ML}}^{a_{i}}\left(n\right)}{\left({ML}^{a_{i}}\left(t\right)-{\hat{ML}}^{a_{i}}\left(t\right)\right)}^{2}),$$

where $\Phi =(\lambda^{a_{2}},\lambda^{a_{3}},\lambda^{a_{4}})$ is the set of parameters to be estimated, and *n* is the size of sample data. This method is implemented by running the command fminsearch from the optimization toolbox in MATLAB. The data of stage 1 and stage 2 are put together, and we only use the reported cumulative test positive cases of stage 2, stage 3 and stage 4 for fitting. The final values predicted by the model in the previous stage were used as the initial values of model in next stage. The initial values and the mean values of estimated parameters and other parameters are shown in Tables 2 and 3, and the fitting results are shown in Fig 8.

**Table 2:** Initial values.

Notation	value	Data Source
*N*	4.561 × 10^6^	[17]
$\left(S_{1}^{a_{3}}\left(0\right);S_{1}^{a_{4}}\left(0\right)\right)$	(259, 588;150, 441)	[17]- [19]
$\left(S_{2}^{a_{2}}\left(0\right);S_{2}^{a_{3}}\left(0\right);S_{2}^{a_{4}}\left(0\right)\right)$	(54,452; 678,063;400,293)	[17]-[19]
$\left(S_{3}^{a_{2}}\left(0\right);S_{3}^{a_{3}}\left(0\right);S_{3}^{a_{4}}\left(0\right)\right)$	(232,986; 708,451; 204,748)	[17]-[19]
$\left(S_{4}^{a_{2}}\left(0\right);S_{4}^{a_{3}}\left(0\right);S_{4}^{a_{4}}\left(0\right)\right)$	(177,698;540,336;156,162)	[17]-[19]
$\left(S_{5}^{a_{2}}\left(0\right);S_{5}^{a_{3}}\left(0\right);S_{5}^{a_{4}}\left(0\right)\right)$	(116,982;355,550;102,757)	[17]-[19]
$\left(S_{6}^{a_{2}}\left(0\right);S_{6}^{a_{3}}\left(0\right);S_{6}^{a_{4}}\left(0\right)\right)$	(85,890;261,172;75,482)	[17]-[19]
$\left(E_{2}^{a_{4}}\left(0\right),E_{3}^{a_{4}}\left(0\right)\right)$	(1,1)	Assumed
$I_{2}^{a_{4}}\left(0\right)$	1	Data analysis

According to the actual situation, initial values of $E_{k}^{a_{i}}\left(0\right)$, $I_{k}^{a_{i}}\left(0\right)$,$Q_{k}^{a_{i}}\left(0\right)$, $H_{k}^{a_{i}}\left(0\right)$ and $R_{k}^{a_{i}}\left(0\right)$ (*k* = 1, 2, ⋯, 6, *i* = 2, 3, 4) that do not appear in the table are 0.

**Table 3:** Parameter values.

Notation	Description	value	Data Source
$\left(\lambda^{a_{2}},\lambda^{a_{3}},\lambda^{a_{4}}\right)$ (Stage 1/2)	Susceptibility of $S_{k}^{a_{i}}$	(0.187,0.2855,0.8153)	LS
$\left(\lambda^{a_{2}},\lambda^{a_{3}},\lambda^{a_{4}}\right)$ (Stage 3)	———–	(0.6919,0.4739,0.3969)	LS
$\left(\lambda^{a_{2}},\lambda^{a_{3}},\lambda^{a_{4}}\right)$ (Stage 4)	———–	(0.0001,0.2845,0.2202)	LS
*μ*_1_	The rate of $E_{k}^{a_{i}}\to I_{k}^{a_{i}}$	$\frac{1}{2.1}$	[31, 32]
$\mu_{2}^{a_{i}}$ (Stage 2/3)	The rate of $E_{k}^{a_{i}}\to I_{k}^{a_{i}}$ without being traced and not detected by nucleic acid testing	$\frac{1}{2.1}\left(1-\varphi^{a_{i}}\right)\left(1-\theta^{a_{i}}\right)$	Caculated
$\left(\theta^{a_{2}},\theta^{a_{3}},\theta^{a_{4}}\right)$ (Stage 2)	The rate of $E_{k}^{a_{i}}$ $\left(I_{k}^{a_{i}}\right)$ being traced and quarantined	(0.1,0.1,0.1)	Assumed
$\left(\theta^{a_{2}},\theta^{a_{3}},\theta^{a_{4}}\right)$ (Stage 3)	———–	(0.37,0.14,0.14)	Assumed
$\left(\theta^{a_{2}},\theta^{a_{3}},\theta^{a_{4}}\right)$ (Stage 3)	———–	(0.53,0.37,0.37)	Derived from [33]
*π* (Stage 2/3)	The rate of $Q_{k}^{a_{i}}\to H_{k}^{a_{i}}$ by nucleic acid testing	1/$\frac{1}{2}$	Data analysis/[34]
*τ* (Stage 2/3)	The rate of $E_{k}^{a_{i}}\to H_{k}^{a_{i}}$($I_{k}^{a_{i}}\to H_{k}^{a_{i}}$) by nucleic acid testing	$\frac{0.8615}{3}$	[35],Data analysis
$\left(\varphi^{a_{2}},\varphi^{a_{3}},\varphi^{a_{4}}\right)$	The rate of $I_{k}^{a_{i}}\to H_{k}^{a_{i}}$ through active medical treatment	(0.067,0.109,0.204)	Data analysis
*γ*_1_	Recovery rate of $I_{k}^{a_{i}}$	0.139	[36]
*γ*_2_	Recovery rate of $H_{k}^{a_{i}}$	$\frac{1}{15}$	[32]

**Fig 9:**
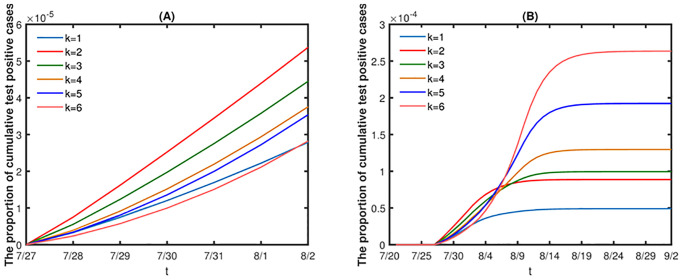
The proportion of cumulative test positive cases with household size *k* in the population with the same household size, i.e. $\frac{I_{k}}{N_{k}}$. The initial values are set as Table 2 and parameter values are set as Table 3.

Based on model fitting results, the proportion of cumulative test positive cases with household size *k* in the population with the same household size was given. According to Fig 9, we can see that the proportions of cumulative test positive cases with household size 2, 3, 4, 5 in the population with same household size are larger in the initial implementation stage of NPIs. With the further strengthening of prevention and control measures, population mainly have contact with their families, so the proportion with large household size is larger in stage 3 and 4.

### The necessity of NPIs

**Fig 10:**
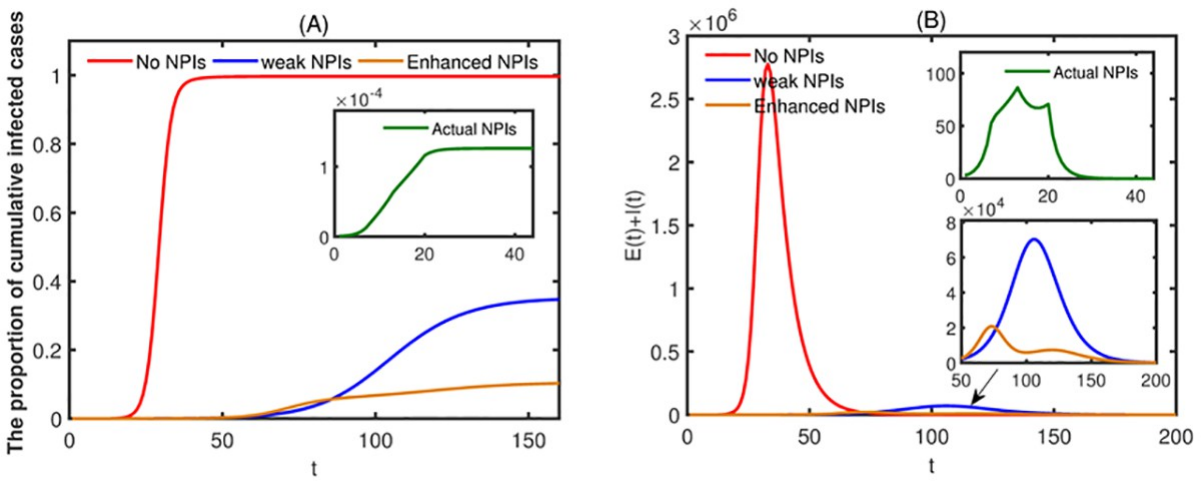
(A) The proportion of cumulative infected cases of all age groups. (B) The number of infected individuals in society. The initial values are set as Table 2 and parameter values are set as Table 3. The red line corresponds to the absence of any NPIs. The green line indicates that the NPIs implemented are the same as those in stage 2 (weak NPIs). The green line indicates the actual implementation of NPIs (strong NPIs).

In the absence of specific drugs, NPIs are effective measures to control the spread of infectious diseases. We approximate the diseases duration as the time when the number of infected individuals in society (*E*(*t*)+ *I*(*t*)) is greater than or equal to 1. From Fig 10 we have that if no NPIs are taken after the outbreak, the diseases duration will be 140 days, the peak value of infected individuals in society will be as high as 2,775,762, the peak value will be reached at *t* = 33, and about 99.7% of population will be infected. If only the same NPIs as in stage 2 are taken after the discovery of the first case (weak NPIs), the diseases duration will be 269 days, the peak value of infected individuals in community will reduce to 70,239, the peak value will be reached at *t* = 106, and about 35.2% of population will be infected. While, if the same NPIs as in stage 2 and stage 3 are taken after the discovery of the first case (enhanced NPIs), the diseases duration will be 327 days, the peak value of infected individuals in community will reduce to 21,821, the peak value will be reached at *t* = 73, and about 10.6% of population will be infected.

### The relationship between the age structure, the household size and the reproduction number

**Fig 11:**
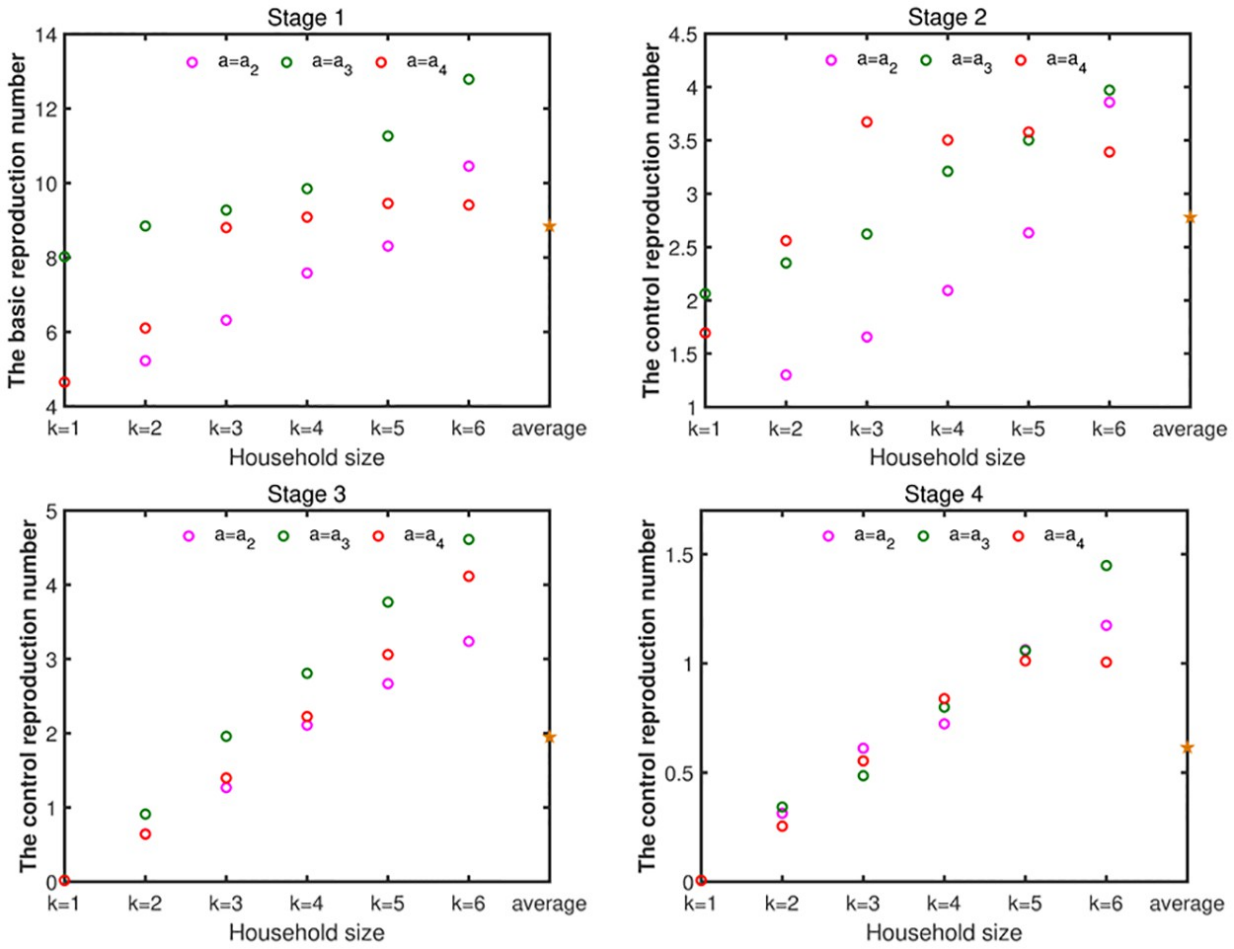
The figures of the basic reproduction number and control reproduction number. The parameters are set as Table 3.

We have obtained the expressions of the basic reproduction number and the control reproduction number in section 3. Combined with the results of the model fitting, the value of the reproduction number can be obtained. For an infectious individual with household size-k, age group-*a*_*i*_, Fig 11 shows that the basic reproduction number ($R_{k,0}^{a_{i}}$) is the biggest in stage 1 and the average value is about 8.84, which means the infectious individual have the strongest transmission ability in this stage. With the implementation of NPIs, the control reproduction numbers $R_{k,c}^{a_{i}}$ in stage 2 is much smaller than the basic reproduction number of stage 1 and the average value is about 2.78, which means the transmission ability of the infectious individual is reduced. In stage 3, the transmission ability of the infectious individual is further weakened, and the control reproduction number $R_{k,c}^{a_{i}}$ is 1.95. In stage 4, the NPIs are the strongest, the transmission ability of the infectious individual is the weakest, and the control reproduction number is the smallest, with an average value of 0.61.

Then, we will study the relationship of reproduction numbers between different age groups when the household sizes are same. In stage 1, for household size smaller than six, the basic reproduction number of the individual with age group-*a*_3_ is the largest, followed by age group-*a*_4_ and age group-*a*_2_ is the smallest, which means an infectious individual with age group-*a*_3_ have the stronger transmission and can cause more infections in stage 1. For the household size 6, the basic reproduction number with age group-*a*_3_ is the largest, followed by age group-*a*_2_. and age group-*a*_4_ is the lowest. Similarly, we can obtain the relationship of the control reproduction number in other stages.

On the whole, for the same age group, as can be seen from the Fig 11, the basic reproduction number and the control reproduction number increase with the increase of the household size at corresponding stage (except $R_{3,c}^{a_{4}}$, $R_{4,c}^{a_{4}}$, $R_{6,c}^{a_{4}}$ in stage 2). That is to say an infectious individual have strong transmission ability and will cause more infections in a large size household.

### The impact of the free transmission stage

In this subsection we will study the impact of the duration of the free transmission stage (stage 1) on COVID-19. The initial values are set as Table 2 and parameters are set as Table 3. We only change the duration of the free transmission stage. From Table 4, we obtain that the shorter the free transmission stage, the smaller the final size of infected individuals, the peak value of daily infected individuals in society (*E*(*t*) + *I*(*t*)) and the peak value of daily quarantine infected individuals and test positive cases (*Q*(*t*) + *H*(*t*)). The free transmission stage of COVID-19 outbreak in Yangzhou in July 2021 lasted for six days and actually infected a total of 570 individuals. If the free transmission stage lasts for 5 days, one day less than the actual situation, the final size would reduce to 335, the peak of daily infected individuals in the community will reduce to 51 and the peak of daily quarantine infected individuals and daily test positive cases will reduce to 202. However, if the free transmission stage lasts for one more day, the three values will reach to 967, 122, 582 respectively. And if the free transmission stage lasts for two more days, the three values will be 1638, 243 and 986, respectively. Thus, the duration of the free transmission stage has great impact on the control of COVID-19 transmission. The longer, the more difficult it is to control the disease, and the greater the burden on medical resources.

**Table 4:** The result of different duration of free transmission stage.

Free transmission stage	Final size	max{*E*(*t*) + *I*(*t*)}	max{*Q*(*t*) + *H*(*t*)}
Five days less	39	6	23
Four days less	67	11	40
Three days less	115	18	69
Two days less	197	31	118
One day less	335	51	202
One more day	967	122	582
Two more days	1638	243	986

Note: $E\left(t\right)+I\left(t\right)=\sum_{k=1}^{6}\sum_{i=1}^{4}\left(E_{k}^{a_{i}}\left(t\right)+I_{k}^{a_{i}}\left(t\right)\right),Q\left(t\right)+H\left(t\right)=\sum_{k=1}^{6}\sum_{i=1}^{4}\left(Q_{k}^{a_{i}}\left(t\right)+H_{k}^{a_{i}}\left(t\right)\right).$

### Impact of random contacts

**Fig 12:**
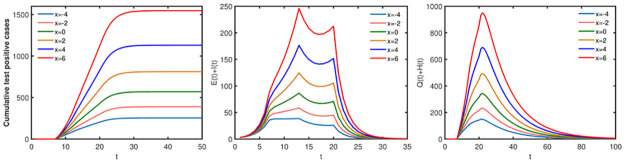
Effects of the number of random contacts on disease transmission. x represents the change in the number of random contacts in each age group, positive numbers represent the increase in random contacts, and negative numbers represent the decrease. The other initial values are set as Table 2 and parameter values are set as Table 3.

**Fig 13:**
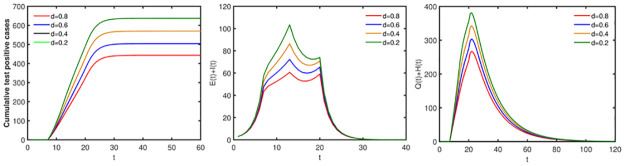
Effects of the proportion of random contacts on disease transmission. The initial values are set as Table 2 and parameter values are set as Table 3.

In this part, we will evaluate the effects of random contacts on the spread of disease. If fix the proportion of random contacts, the greater the number of random contacts, the more adverse the disease control. And the final size, the peak value of infected individuals in society (*E*(*t*)+ *I*(*t*)) and the peak value of quarantine infected individuals and nucleic acid test positive individuals (*Q*(*t*)+ *H*(*t*)) increase as the number of random contacts increases (see Fig 12). In addition, we take the proportion of random contacts for all groups as the same value and represent it with *d* in the first two stages (the proportion of the regular contacts is 1 − *d*), and the proportion of random contacts is also 0.05 in stage 3 and stage 4. Then from Fig 13 we have that if fix the number of random contacts, the final size, the peak value of infected individuals in society (E(t) + I(t)) and the peak value of quarantine infected individuals and nucleic acid test positive individuals (Q(t) + H(t)) decrease as the proportion of random contacts increases.

**Fig 14:**
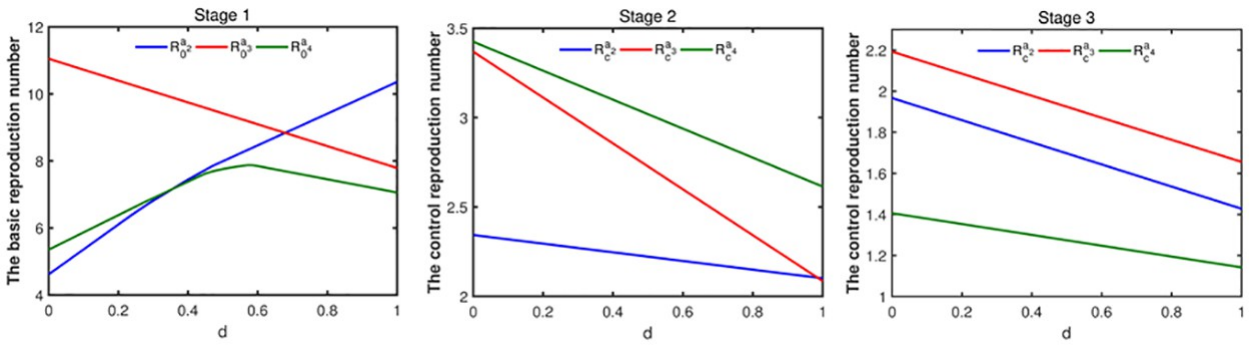
Effects of the proportion of random contacts on the basic reproduction number and control reproduction number. The parameter values are set as Table 3.

While from Fig 14, we can see that the basic reproduction number of individuals with age group-*a*_2_ increases with increasing of the portion of random contacts. For the individuals with age group-*a*_3_, with increasing of the portion of random contacts, the basic reproduction number decreases. While for the individuals with age group-*a*_4_, when the portion of random contacts is less than or equal to 0.58, the basic reproduction number increases with the increase of the proportion of random contacts; when the portion of random contacts is larger than 0.58, the basic reproduction number decreases with the increase of the proportion of random contacts. That is to say, for individuals with age group-*a*_2_, increasing the proportion of random contact will increase the ability to transmit disease and is not conducive to disease prevention and control. For individuals with age group *a*_3_, increasing the proportion of random contact will reduce the ability to transmit diseases, which is conducive to disease prevention and control. As for all individuals, the control reproduction number decreases with the increase of the proportion of random contacts in stage 2, stage 3 and stage 4, which means that reducing cluster transmission is conducive to disease control.

### The impact of summer vacation

**Fig 15:**
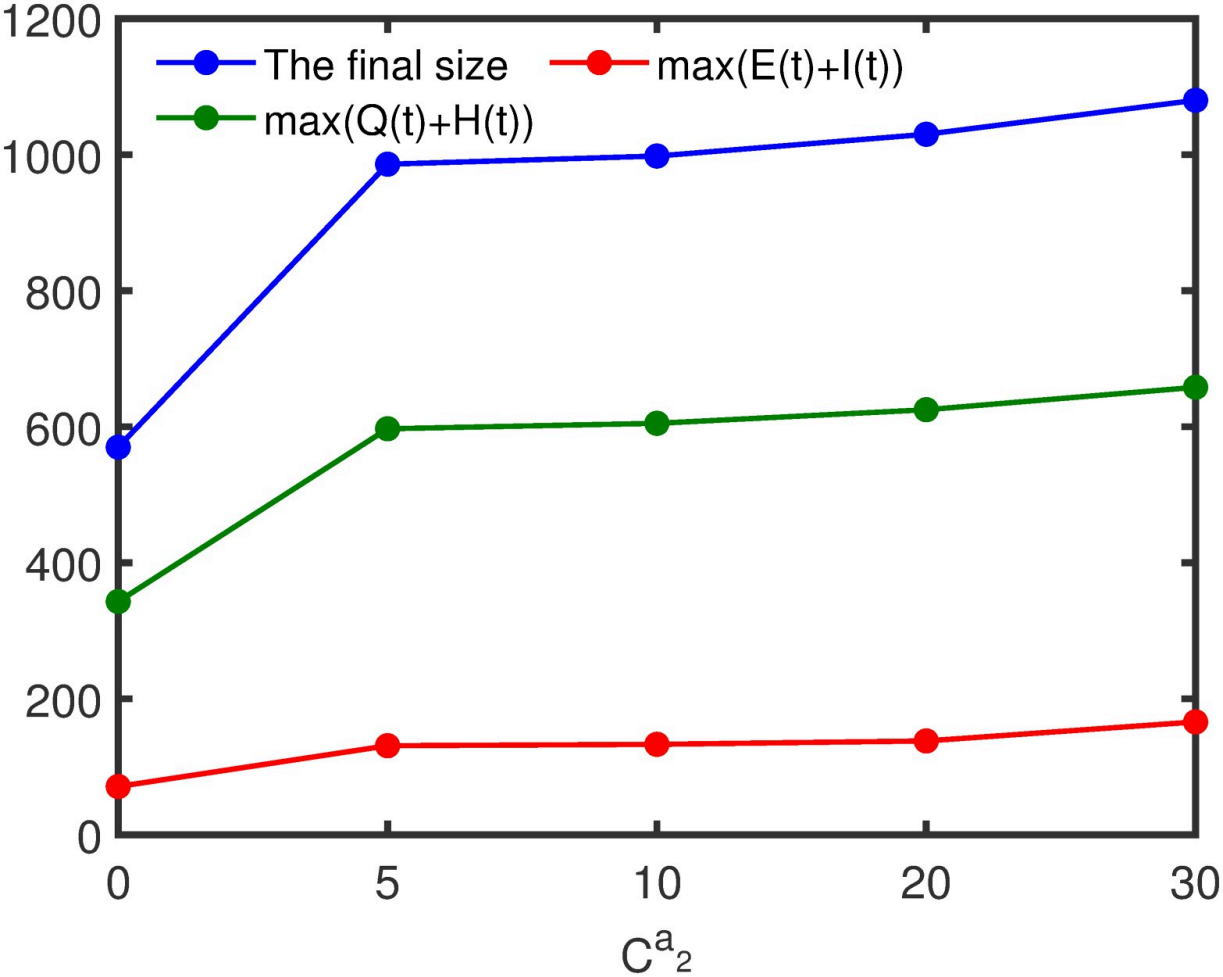
The influence of summer vacation on the spread of COVID-19. Initial values are set as Table 2 and parameters are set as Table 3. $qq=\frac{2}{3}$ and the horizontal axis represents the number of regular contacts between students among age group-*a*_2_ at school during daytime.

Schools are highly densely populated places and are prone to all kinds of infectious diseases. If COVID-19 breaks out in the spring or autumn semester, it will bring more difficulties to the control of the epidemic. During daytime, we increase the regular contacts between students among age group-*a*_2_ and ignore the regular contacts between these students and the other individuals. In addition, we assume that the proportion of *qq* seniors who had regular contact with students in summer vacation will generate regular contact at the activity place during daytime. Based on the contact matrix diagram (Fig 6), the number of contacts made at school of individuals with age group *a*_2_ is 5.304. Assuming that the number of random contact is 0.304, the number of regular contact between students is 5. The number of contacts made at school of individuals with age group *a*_3_ is 0.12. And assume that 0.12 is the number of random contacts. Then, we have $X^{a_{2}}=6.544,X^{a_{3}}=4.99,X^{a_{4}}=4.33$. Through model simulation and combined with Fig 15, we get that when the number of regular contacts of students among age group *a*_2_ is 0, that is the students are on summer vacation, the final size, the peak value of infected individuals in community(*E*(*t*) + *I*(*t*)) and the peak value of quarantine infected individuals and nucleic acid test positive individuals (*Q*(*t*) + *H*(*t*)) are 570, 71, 343 respectively. While, when the number of regular contacts of students among age group *a*_2_ is 5, the three values are as high as 986, 131, 597 respectively. However, the susceptibility of individuals with age *a*_2_ is the lowest. Thus, these three numbers did not increase much as the number of regular contacts increase.

### The comparison of heterogeneous mixing and contact patterns with homogeneous case

**Fig 16:**
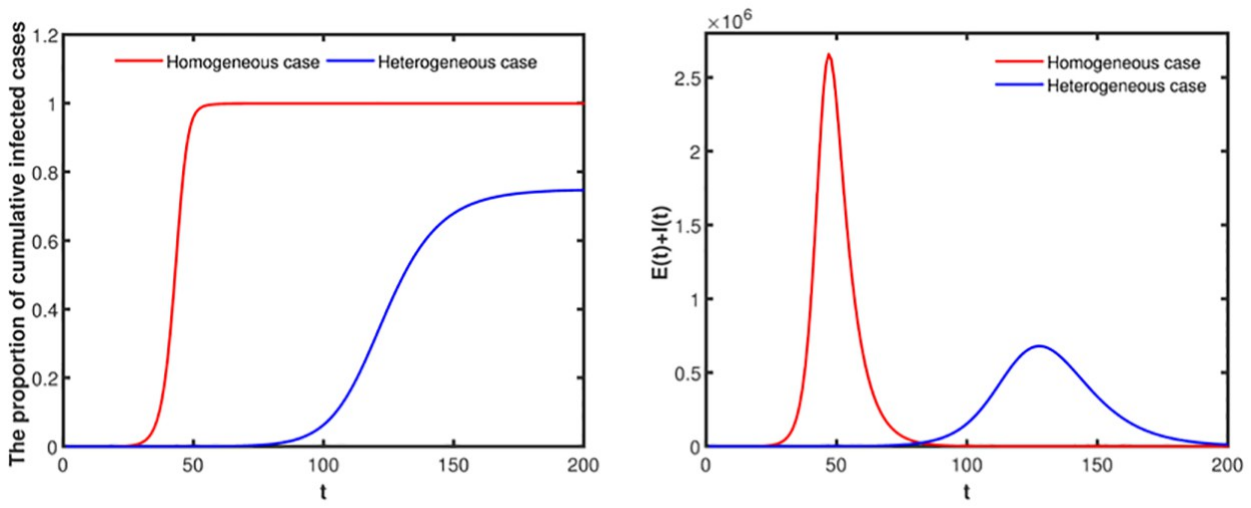
The impact of heterogeneous and homogeneous mixing and contact patterns on disease transmission without NPIs.

In this section, we will study the impact of heterogeneous and homogeneous mixing and contact patterns on disease transmission without NPIs. We set $\lambda^{a_{2}}=0.0374$, $\lambda^{a_{3}}=0.0571$, $\lambda^{a_{2}}=0.1631$. The initial values are set as Table 2 and The other parameters are set as Table 3. The susceptibility and the number of contacts in homogeneous case is the weighted average of susceptibility in heterogeneous case. It is not difficult to see from Fig 16 that homogenous mixing and contact patterns may overestimate the actual disease transmission.

## Conclusion and discussion

Human contact behavior is a key factor in the spread of infectious diseases. For each of us, we will have regular contacts with our colleagues, classmates, family members and so on at a fixed time during daytime. In addition, we also have random contacts with individuals for a short time at short distances in some leisure and entertainment places or on the way to work/school or on the way home. In other words, contacts that occur during daytime include regular contacts and random contacts. At night, we have regular contacts with our families at home. Considering the two contact patterns, we have to consider age heterogeneity and household structure. It is an extremely complex task. However, the work is of great significance, which can more truly reflect the contact patterns of population, and provide reasonable suggestions for disease prevention and control.

Usually before the first case is diagnosed, the disease spreads freely among population, and the duration of this stage has an important impact on subsequent disease control. In this paper, we show that the shorter the duration of the free transmission stage, the sooner the infected individuals in the population will be discovered, and the more conducive to disease control. If it had been discovered one day earlier, the final size would have been reduced by 235. However, if it had been discovered one day later, the final size would have been increased by 397. These reflects the necessity of regular nucleic acid testing under the dynamic zero-COVID policy. At the same time, studies have shown that if no NPIs is taken, about 99.7% of population will be infected. If weak NPIs are taken, then about 35.2% of population will be infected. While, if enhanced NPIs are taken, then about 10.6% of population will be infected.

Furthermore, we found that the larger the number of random contacts, the more detrimental to disease control. And the final size, the peak value of infected individuals in society (*E*(*t*) + *I*(*t*)) and the peak value of quarantine infected individuals and nucleic acid test positive individuals (*Q*(*t*) + *H*(*t*)) increase as the number of random contacts increases. In stage 1 (free transmission stage), if the number of random contacts is invariable, for individuals with age group-*a*_2_ decreasing the proportion of random contacts was beneficial for disease control. For individuals with age group-*a*_3_, increasing the proportion of random contacts was beneficial for disease control. While for the individuals with age group-*a*_4_, when the portion of random contacts is less than or equal to 0.58, decreasing the proportion of random contact is beneficial for disease control; when the portion of random contacts is larger than 0.58, increasing the proportion of random contact is beneficial for disease control. In addition, we also found that an infectious individuals will lead the most infections in stage 1. As the implementation of NPIs, the ability of the infectious individual to transmit the disease decreases in stage 2 and stage 3. In stage 4, NPIs are strongest and the transmission ability of the infectious individual is weakest, and then the disease will decline in the population. As for the individuals with the same age group, an infectious individual will cause more infections in a household with big size. Then, we assessed the impact of summer vacation on the spread of COVID-19. Once the epidemic occurs in the spring or autumn semester, there will be a large-scale increase in both the final size and the peak value of infected individuals. Finally, through model simulation we have that homogenous mixing and contact patterns may overestimate the actual spread of the disease.

However, the study is subject to a number of limitations. First, in order to facilitate the study, we do not consider the quarantine of susceptible individuals. Second, in modeling, we did not take into account the changes in household structure brought about by quarantine the infected individuals. When the number of infected individuals is relatively small, it has little impact on the follow-up research and analysis. However, when the number of infected individuals is large, the impact can not be ignored. In this case, we need to add the evolution equation of each household structure to the model.

## Supporting information

S1 File Appendix. (PDF)
