# Supplementary material for: Assessing the impact of different contact patterns on disease transmission: Taking COVID-19 as a case
Source: PLoS One. 2024 Apr 11;19(4):e0300884. doi: 10.1371/journal.pone.0300884 (PMC11008907; doi:10.1371/journal.pone.0300884)
Supplement: S1 File — (PDF) [file pone.0300884.s001.pdf]

$i = 2$:

$$
\begin{cases}
\alpha_2^{a_2}(t) & = 1 - \frac{hF_2^{a_2a_3}}{N_2^{a_2}}(1-\lambda^{a_2}\bar{I}_2^{a_3}(t)) - \frac{F_2^{a_2a_4}}{N_2^{a_2}}(1-\lambda^{a_2}\bar{I}_2^{a_4}(t)) \\
& -c(1-h)\frac{F_2^{a_2a_3}}{N_2^{a_2}}(1-\lambda^{a_2}\bar{I}_1^{a_4}(t)) - (1-c)(1-h)\frac{F_2^{a_2a_3}}{N_2^{a_2}}(1-\lambda^{a_2}\bar{I}_2^{a_4}(t))^2, \\
\alpha_3^{a_2}(t) & = 1 - \frac{hF_3^{a_2a_3a_3}}{N_3^{a_2}}(1-\lambda^{a_2}\bar{I}_3^{a_3}(t)) - \frac{c(1-h)F_3^{a_2a_3a_3}}{N_3^{a_2}}(1-\lambda^{a_2}\bar{I}_1^{a_4}(t)) \\
& -\frac{(1-c)(1-h)F_3^{a_2a_3a_3}}{N_3^{a_2}}(1-\lambda^{a_2}\bar{I}_2^{a_4}(t))^2 - \frac{F_3^{a_2a_4a_4}}{N_3^{a_2}}(1-\lambda^{a_2}\bar{I}_3^{a_4}(t))^2 \\
& -\frac{hF_3^{a_1a_2a_3}}{N_3^{a_2}}(1-\lambda^{a_2}\bar{I}_3^{a_1}(t))(1-\lambda^{a_2}\bar{I}_3^{a_3}(t)) - \frac{c(1-h)F_3^{a_1a_2a_3}}{N_3^{a_2}}(1-\lambda^{a_2}\bar{I}_3^{a_1}(t)) \\
& (1-\lambda^{a_2}\bar{I}_1^{a_4}(t)) - \frac{(1-c)(1-h)F_3^{a_1a_2a_3}}{N_3^{a_2}}(1-\lambda^{a_2}\bar{I}_3^{a_1}(t))(1-\lambda^{a_2}\bar{I}_2^{a_4}(t))^2, \\
\alpha_4^{a_2}(t) & = 1 - 2\frac{hF_4^{a_2a_2a_3a_3}}{N_4^{a_2}}(1-\lambda^{a_2}\bar{I}_4^{a_2}(t))(1-\lambda^{a_2}\bar{I}_4^{a_3}(t)) - 2\frac{c(1-h)F_4^{a_2a_2a_3a_3}}{N_4^{a_2}}(1-\lambda^{a_2}\bar{I}_4^{a_2}(t)) \\
& (1-\lambda^{a_2}\bar{I}_1^{a_4}(t)) - 2\frac{(1-c)(1-h)F_4^{a_2a_2a_3a_3}}{N_4^{a_2}}(1-\lambda^{a_2}\bar{I}_4^{a_2}(t))(1-\lambda^{a_2}\bar{I}_2^{a_4}(t))^2 \\
& -\frac{hF_4^{a_1a_2a_3a_3}}{N_4^{a_2}}(1-\lambda^{a_2}\bar{I}_4^{a_1}(t))(1-\lambda^{a_2}\bar{I}_4^{a_3}(t)) - \frac{c(1-h)F_4^{a_1a_2a_3a_3}}{N_4^{a_2}}(1-\lambda^{a_2}\bar{I}_4^{a_1}(t)) \\
& (1-\lambda^{a_2}\bar{I}_1^{a_4}(t)) - \frac{(1-c)(1-h)F_4^{a_1a_2a_3a_3}}{N_4^{a_2}}(1-\lambda^{a_2}\bar{I}_4^{a_1}(t))(1-\lambda^{a_2}\bar{I}_2^{a_4}(t))^2 \\
& -\frac{F_4^{a_2a_3a_3a_3}}{N_4^{a_2}}(1-\lambda^{a_2}\bar{I}_4^{a_3}(t)) - \frac{F_4^{a_2a_3a_3a_4}}{N_4^{a_2}}(1-\lambda^{a_2}\bar{I}_4^{a_4}(t)) - \frac{F_4^{a_2a_3a_4a_4}}{N_4^{a_2}}(1-\lambda^{a_2}\bar{I}_4^{a_4}(t))^2, \\
\alpha_5^{a_2}(t) & = 1 - \frac{F_5^{a_1a_2a_3a_3a_4}}{N_5^{a_2}}(1-\lambda^{a_2}\bar{I}_5^{a_1}(t))(1-\lambda^{a_2}\bar{I}_5^{a_4}(t)) - \frac{F_5^{a_2a_3a_3a_3a_3}}{N_5^{a_2}}(1-\lambda^{a_2}\bar{I}_5^{a_3}(t)) \\
& -2\frac{F_5^{a_2a_2a_3a_3a_3}}{N_5^{a_2}}(1-\lambda^{a_2}\bar{I}_5^{a_2}(t))(1-\lambda^{a_2}\bar{I}_5^{a_3}(t)) - \frac{F_5^{a_2a_3a_3a_3a_4}}{N_5^{a_2}}(1-\lambda^{a_2}\bar{I}_5^{a_4}(t)) \\
& -2\frac{F_5^{a_2a_2a_3a_3a_4}}{N_5^{a_2}}(1-\lambda^{a_2}\bar{I}_5^{a_2}(t))(1-\lambda^{a_2}\bar{I}_5^{a_4}(t)) - \frac{F_5^{a_2a_3a_3a_4a_4}}{N_5^{a_2}}(1-\lambda^{a_2}\bar{I}_5^{a_4}(t))^2, \\
\alpha_6^{a_2}(t) & = 1 - \frac{F_6^{a_1a_2a_3a_3a_4a_4}}{N_6^{a_2}}(1-\lambda^{a_2}\bar{I}_6^{a_1}(t))(1-\lambda^{a_2}\bar{I}_6^{a_4}(t))^2 - 2\frac{F_6^{a_2a_2a_3a_3a_3a_4}}{N_6^{a_2}} \\
& (1-\lambda^{a_2}\bar{I}_6^{a_2}(t))(1-\lambda^{a_2}\bar{I}_6^{a_4}(t)) - 2\frac{F_6^{a_2a_2a_3a_3a_4a_4}}{N_6^{a_2}}(1-\lambda^{a_2}\bar{I}_6^{a_2}(t))(1-\lambda^{a_2}\bar{I}_6^{a_4}(t))^2 \\
& -2\frac{F_6^{a_2a_2a_3a_3a_3a_3}}{N_6^{a_2}}(1-\lambda^{a_2}\bar{I}_6^{a_2}(t))(1-\lambda^{a_2}\bar{I}_6^{a_3}(t)) - \frac{F_6^{a_2a_3a_3a_3a_4a_4}}{N_6^{a_2}}(1-\lambda^{a_2}\bar{I}_6^{a_4}(t))^2 \\
& -(1-q)\frac{F_6^{a_2a_3a_3a_4a_4a_4}}{N_6^{a_2}}(1-\lambda^{a_2}\bar{I}_6^{a_4}(t))^3 - q\frac{F_6^{a_2a_3a_3a_4a_4a_4}}{N_6^{a_2}}(1-\lambda^{a_2}\bar{I}_6^{a_4}(t))^2.
\end{cases}
$$

$i = 3$:

$$\begin{cases}
\alpha_1^{a_3}(t) & = 1 - P(L_1^{a_3}(t) = WO) \sum_r P(C_1^{a_3}(t) = r | L_1^{a_3}(t) = WO) \prod_{k'=1}^{6} (1 - \lambda^{a_3} \bar{I}_{k'}^{a_3}(t))^{g(k',r)}, \\
\alpha_2^{a_3}(t) & = 1 - h \left(\frac{F_2^{a_1 a_3}}{N^{a_3}} (1 - \lambda^{a_3} \bar{I}_2^{a_1}(t)) + \frac{F_2^{a_2 a_3}}{N^{a_3}} (1 - \lambda^{a_3} \bar{I}_2^{a_2}(t)) \right) - P(L_2^{a_3}(t) = WO) \\
& \quad \sum_r P(C_2^{a_3}(t) = r | L_2^{a_3}(t) = WO) \prod_{k'=1}^{6} (1 - \lambda^{a_3} \bar{I}_{k'}^{a_3}(t))^{g(k',r)}, \\
\alpha_3^{a_3}(t) & = 1 - h \frac{F_3^{a_1 a_3 a_3}}{N_3^{a_3}} (1 - \lambda^{a_3} \bar{I}_3^{a_1}(t)) - h \frac{F_3^{a_2 a_3 a_3}}{N_3^{a_3}} (1 - \lambda^{a_3} \bar{I}_3^{a_2}(t)) \\
& \quad - h \frac{F_3^{a_1 a_2 a_3}}{N_3^{a_3}} (1 - \lambda^{a_3} \bar{I}_3^{a_1}(t))(1 - \lambda^{a_3} \bar{I}_3^{a_2}(t)) \\
& \quad - P(L_3^{a_3}(t) = WO) \sum_r P(C_3^{a_3}(t) = r | L_3^{a_3}(t) = WO) \prod_{k'=1}^{6} (1 - \lambda^{a_3} \bar{I}_{k'}^{a_3}(t))^{g(k',r)}, \\
\alpha_4^{a_3}(t) & = 1 - h \left(\frac{F_4^{a_2 a_2 a_3 a_3}}{N_4^{a_3}} (1 - \lambda^{a_3} \bar{I}_4^{a_2}(t))^2 + \frac{F_4^{a_1 a_2 a_3 a_3}}{N_4^{a_3}} (1 - \lambda^{a_3} \bar{I}_4^{a_1}(t))(1 - \lambda^{a_3} \bar{I}_4^{a_2}(t)) \right) \\
& \quad - \frac{F_4^{a_2 a_3 a_3 a_3}}{N_4^{a_3}} (1 - \lambda^{a_3} \bar{I}_4^{a_2}(t)) - P(L_4^{a_3}(t) = WO) \sum_r P(C_4^{a_3}(t) = r | L_4^{a_3}(t) = WO) \\
& \quad \prod_{k'=1}^{6} (1 - \lambda^{a_3} \bar{I}_{k'}^{a_3}(t))^{g(k',r)}, \\
\alpha_5^{a_3}(t) & = 1 - \frac{F_5^{a_1 a_3 a_3 a_3 a_3}}{N_5^3} (1 - \lambda^{a_3} \bar{I}_5^{a_1}(t)) - \frac{F_5^{a_2 a_3 a_3 a_3 a_3}}{N_5^3} (1 - \lambda^{a_3} \bar{I}_5^{a_2}(t)) \\
& \quad - \frac{F_5^{a_2 a_2 a_3 a_3 a_3}}{N_5^3} (1 - \lambda^{a_3} \bar{I}_5^{a_2}(t))^2 - P(L_5^{a_3}(t) = WO) \sum_r P(C_5^{a_3}(t) = r | L_5^{a_3}(t) = WO) \\
& \quad \prod_{k'=1}^{6} (1 - \lambda^{a_3} \bar{I}_{k'}^{a_3}(t))^{g(k',r)}, \\
\alpha_6^{a_3}(t) & = 1 - \frac{F_6^{a_2 a_2 a_3 a_3 a_3 a_3}}{N_6^{a_3}} (1 - \lambda^{a_3} \bar{I}_6^{a_2}(t))^2 - \frac{F_6^{a_1 a_3 a_3 a_3 a_3 a_3}}{N_6^{a_3}} (1 - \lambda^{a_3} \bar{I}_6^{a_1}(t)) \\
& \quad - P(L_6^{a_3}(t) = WO) \sum_r P(C_6^{a_3}(t) = r | L_6^{a_3}(t) = WO) \prod_{k'=1}^{6} (1 - \lambda^{a_3} \bar{I}_{k'}^{a_3}(t))^{g(k',r)}.
\end{cases}$$

The second item on the right of the first expression indicates the probability of not being infected of a susceptible individual with household size-1, age group-a_3 in the workplace. $g(k', r) = \left[r \frac{N_{k'}^{a_3} - \Delta_{k'}^{a_3}}{N^{a_3} - \Delta^{a_3}} \right]$ represents the number of individuals with household size-k', age group-a_3 among the r regular contacts. $\Delta_{k'}^{a_3}$ represents the number of unemployed individuals with household size-k', age group a_3. Δ^{a_3} represents the number of unemployed individuals with age group a_3, which can be expressed as:

$$\Delta^{a_3} = h(F_2^{a_1 a_3} + F_2^{a_2 a_3} + F_3^{a_1 a_3 a_3} + F_3^{a_2 a_3 a_3} + F_3^{a_1 a_2 a_3} + F_4^{a_1 a_2 a_3 a_3} + F_4^{a_2 a_2 a_3 a_3}) + F_4^{a_1 a_3 a_3 a_3}$$

$$+ F_4^{a_2 a_3 a_3 a_3} + F_5^{a_1 a_3 a_3 a_3 a_3} + F_5^{a_2 a_3 a_3 a_3 a_3} + F_5^{a_2 a_2 a_3 a_3 a_3} + F_6^{a_1 a_3 a_3 a_3 a_3 a_3} + F_6^{a_2 a_2 a_3 a_3 a_3 a_3}.$$

The second term on the right of the second formula indicates the probability of not being infected of a susceptible individual with household size-2, age group-a_3 who has regular contacts with an infectious individual with age group-a_1 or age group-a_2 in the same household, which can be derived as follows:

$$P(L_2^{a_3}(t) = HO) P(C_2^{a_3}(t) = 1 | L_2^{a_3}(t) = HO) P(\text{Uninfected during daytime} | \mathrm{C_2^{a_3}(t) = 1, L_2^{a_3}(t) = HO})$$

$$= \frac{h(F_2^{a_1 a_3} + F_2^{a_2 a_3})}{N_2^{a_3}} \times 1 \times$$
$$\left(\frac{F_2^{a_1 a_3}}{h(F_2^{a_1 a_3} + F_2^{a_2 a_3})} (1 - \lambda^{a_3} \bar{I}_2^{a_1}(t)) + \frac{F_2^{a_2 a_3}}{h(F_2^{a_1 a_3} + F_2^{a_2 a_3})} (1 - \lambda^{a_3} \bar{I}_2^{a_2}(t)) \right)$$
$$= \frac{h F_2^{a_1 a_3}}{N_2^{a_3}} (1 - \lambda^{a_3} \bar{I}_2^{a_1}(t)) + \frac{h F_2^{a_2 a_3}}{N_2^{a_3}} (1 - \lambda^{a_3} \bar{I}_2^{a_2}(t)).$$

During daytime, individuals in age group-a_4 either stay at home alone, or have regular contact with individuals in age group-a_1 or age group-a_2 or age group-a_4. So $i = 4$:

$$
\left\{
\begin{aligned}
\alpha_1^{a_4}(t) &= 1 - \tfrac{c(1-h)F_2^{a_1a_3}}{N_1^{a_4}}(1 - \lambda^{a_4}\bar{I}_2^{a_1}(t)) - \tfrac{c(1-h)F_3^{a_1a_3a_3}}{N_1^{a_4}}(1 - \lambda^{a_4}\bar{I}_3^{a_1}(t)) \\
&\quad - \tfrac{c(1-h)F_3^{a_1a_2a_3}}{N_1^{a_4}}(1 - \lambda^{a_4}\bar{I}_3^{a_1}(t))(1 - \lambda^{a_4}\bar{I}_3^{a_2}(t)) - \tfrac{c(1-h)F_4^{a_1a_2a_3a_3}}{N_1^{a_4}}(1 - \lambda^{a_4}\bar{I}_4^{a_1}(t)) \\
&\quad (1 - \lambda^{a_4}\bar{I}_4^{a_2}(t)) - c(1-h)\tfrac{F_2^{a_2a_3}}{N_1^{a_4}}(1 - \lambda^{a_4}\bar{I}_2^{a_2}(t)) - \tfrac{c(1-h)F_3^{a_2a_3a_3}}{N_1^{a_4}}(1 - \lambda^{a_4}\bar{I}_3^{a_2}(t)) \\
&\quad - \tfrac{c(1-h)F_4^{a_2a_2a_3a_3}}{N_1^{a_4}}(1 - \lambda^{a_4}\bar{I}_4^{a_2}(t))^2 - (1-q)\Gamma_1^{a_4} - q\Gamma_1^{a_4}\textstyle\prod_{k'=1}^{6}(1 - \lambda^{a_4}\bar{I}_{k'}^{a_4}(t))^{\psi(k',r)}, \\
\alpha_2^{a_4}(t) &= 1 - \tfrac{F_2^{a_1a_4}}{N_2^{a_4}}(1 - \lambda^{a_4}\bar{I}_2^{a_1}(t)) - \tfrac{F_2^{a_2a_4}}{N_2^{a_4}}(1 - \lambda^{a_4}\bar{I}_2^{a_2}(t) - 2\tfrac{F_2^{a_4a_4}}{N_2^{a_4}}(\tfrac{(1-c)(1-h)F_2^{13}}{F_2^{a_4a_4}} \\
&\quad (1 - \lambda^{a_4}\bar{I}_2^{a_1}(t)))(1 - \lambda^{a_4}\bar{I}_2^{a_4}(t)) + \tfrac{(1-c)(1-h)F_2^{a_2a_3}}{F_2^{a_4a_4}}(1 - \lambda^{a_4}\bar{I}_2^{a_2}(t))(1 - \lambda^{a_4}\bar{I}_2^{a_4}(t)) \\
&\quad + \tfrac{(1-c)(1-h)F_3^{a_1a_3a_3}}{F_2^{a_4a_4}}(1 - \lambda^{a_4}\bar{I}_3^{a_1}(t))(1 - \lambda^{a_4}\bar{I}_2^{a_4}(t)) + \tfrac{(1-c)(1-h)F_3^{a_2a_3a_3}}{F_2^{a_4a_4}} \\
&\quad (1 - \lambda^{a_4}\bar{I}_3^{a_2}(t))(1 - \lambda^{a_4}\bar{I}_2^{a_4}(t)) + \tfrac{(1-c)(1-h)F_3^{a_1a_2a_3}}{F_2^{a_4a_4}}(1 - \lambda^{a_4}\bar{I}_3^{a_1}(t)) \\
&\quad (1 - \lambda^{a_4}\bar{I}_3^{a_2}(t))(1 - \lambda^{a_4}\bar{I}_2^{a_4}(t)) + \tfrac{(1-c)(1-h)F_4^{a_1a_2a_3a_3}}{F_2^{a_4a_4}}(1 - \lambda^{a_4}\bar{I}_4^{a_1}(t)) \\
&\quad (1 - \lambda^{a_4}\bar{I}_4^{a_2}(t))(1 - \lambda^{a_4}\bar{I}_2^{a_4}(t)) + \tfrac{(1-c)(1-h)F_4^{a_2a_2a_3a_3}}{F_2^{a_4a_4}}(1 - \lambda^{a_4}\bar{I}_4^{a_2}(t))^2 \\
&\quad (1 - \lambda^{a_4}\bar{I}_2^{a_4}(t))) - (1-q)\tfrac{F_2^{a_3a_4}}{N_2^{a_4}} - (1-q)(\Gamma_2^{a_4} - \tfrac{F_2^{a_3a_4}}{N_2^{a_4}})(1 - \lambda^{a_4}\bar{I}_4^{a_2}(t)) \\
&\quad - q\Gamma_2^{a_4}\textstyle\prod_{k'=1}^{6}(1 - \lambda^{a_4}\bar{I}_{k'}^{a_4}(t))^{\psi(k',r)}, \\
\alpha_3^{a_4}(t) &= 1 - 2\tfrac{F_3^{a_1a_4a_4}}{N_3^{a_4}}(1 - \lambda^{a_4}\bar{I}_3^{a_1}(t))(1 - \lambda^{a_4}\bar{I}_3^{a_4}(t)) - (1-q)\tfrac{F_3^{a_3a_3a_4}}{N_3^{a_4}} \\
&\quad - 2\tfrac{F_3^{a_2a_4a_4}}{N_3^{a_4}}(1 - \lambda^{a_4}\bar{I}_3^{a_2}(t))(1 - \lambda^{a_4}\bar{I}_3^{a_4}(t)) - 2(1-q)\tfrac{F_3^{a_3a_4a_4}}{N_3^{a_4}}(1 - \lambda^{a_4}\bar{I}_3^{a_4}(t)) \\
&\quad - q\Gamma_3^{a_4}\textstyle\prod_{k'=1}^{6}(1 - \lambda^{a_4}\bar{I}_{k'}^{a_4}(t))^{\psi(k',r)}, \\
\alpha_4^{a_4}(t) &= 1 - \tfrac{F_4^{a_2a_3a_3a_4}}{N_4^{a_4}}(1 - \lambda^{a_4}\bar{I}_4^{a_2}(t)) - 2\tfrac{F_4^{a_3a_3a_4a_4}}{N_4^{a_4}}(1 - \lambda^{a_4}\bar{I}_4^{a_4}(t)) - 2\tfrac{F_4^{a_1a_3a_4a_4}}{N_4^{a_4}} \\
&\quad (1 - \lambda^{a_4}\bar{I}_4^{a_1}(t))(1 - \lambda^{a_4}\bar{I}_4^{a_4}(t)) - 2\tfrac{F_4^{a_2a_3a_4a_4}}{N_4^{a_4}}(1 - \lambda^{a_4}\bar{I}_4^{a_2}(t))(1 - \lambda^{a_4}\bar{I}_4^{a_4}(t)) \\
&\quad - (1-q)\tfrac{F_4^{a_3a_3a_3a_4} + 2F_4^{a_3a_3a_4a_4}(1-\lambda^{a_4}\bar{I}_4^{a_4}(t))}{N_4^{a_4}} \\
&\quad - q\Gamma_4^{a_4}\textstyle\prod_{k'=1}^{6}(1 - \lambda^{a_4}\bar{I}_{k'}^{a_4}(t))^{\psi(k',r)}, \\
\alpha_5^{a_4}(t) &= 1 - \tfrac{F_5^{a_2a_3a_3a_3a_4}}{N_5^{a_4}}(1 - \lambda^{a_4}\bar{I}_5^{a_2}(t)) - \tfrac{F_5^{a_1a_2a_3a_3a_4}}{N_5^{a_4}}(1 - \lambda^{a_4}\bar{I}_5^{a_1}(t))(1 - \lambda^{a_4}\bar{I}_5^{a_2}(t)) \\
&\quad - \tfrac{F_5^{a_2a_2a_3a_3a_4}}{N_5^{a_4}}(1 - \lambda^{a_4}\bar{I}_5^{a_2}(t))^2 - 2\tfrac{F_5^{a_2a_3a_3a_4a_4}}{N_5^{a_4}}(1 - \lambda^{a_4}\bar{I}_5^{a_2}(t))(1 - \lambda^{a_4}\bar{I}_5^{a_4}(t)) \\
&\quad - (1-q)\Gamma_5^{a_4}\tfrac{F_5^{a_3a_3a_3a_3a_4} + 2F_5^{a_3a_3a_3a_4a_4}(1-\lambda^{a_4}\bar{I}_5^{a_4}(t)) + 3F_5^{a_3a_3a_4a_4a_4}(1-\lambda^{a_4}\bar{I}_5^{a_4}(t))^2}{N_5^{a_4}} \\
&\quad - q\Gamma_5^{a_4}\textstyle\prod_{k'=1}^{6}(1 - \lambda^{a_4}\bar{I}_{k'}^{a_4}(t))^{\psi(k',r)} \\
\alpha_6^{a_4}(t) &= 1 - 2\tfrac{F_6^{a_1a_1a_3a_3a_4a_4}}{N_6^{a_4}}(1 - \lambda^{a_4}\bar{I}_6^{a_1}(t))^2(1 - \lambda^{a_4}\bar{I}_6^{a_4}(t)) - 2\tfrac{F_6^{a_1a_2a_3a_3a_4a_4}}{N_6^{a_4}}(1 - \lambda^{a_4}\bar{I}_6^{a_1}(t)) \\
&\quad (1 - \lambda^{a_4}\bar{I}_6^{a_2}(t))(1 - \lambda^{a_4}\bar{I}_6^{a_4}(t)) - 2\tfrac{F_6^{a_2a_2a_3a_3a_4a_4}}{N_6^{a_4}}(1 - \lambda^{a_4}\bar{I}_6^{a_2}(t))^2(1 - \lambda^{a_4}\bar{I}_6^{a_4}(t)) \\
&\quad - \tfrac{F_6^{a_2a_2a_3a_3a_3a_4}}{N_6^{a_4}}(1 - \lambda^{a_4}\bar{I}_6^{a_2}(t))^2 - 2\tfrac{F_6^{a_2a_3a_3a_3a_4a_4}}{N_6^{a_4}}(1 - \lambda^{a_4}\bar{I}_6^{a_2}(t))(1 - \lambda^{a_4}\bar{I}_6^{a_4}(t)) \\
&\quad - 2\tfrac{F_6^{a_2a_3a_3a_4a_4a_4}}{N_6^{a_4}}(1 - \lambda^{a_4}\bar{I}_6^{a_2}(t))(1 - \lambda^{a_4}\bar{I}_6^{a_4}(t)) - (1-q)\tfrac{F_6^{a_2a_3a_3a_4a_4a_4}}{N_6^{a_4}} \\
&\quad (1 - \lambda^{a_4}\bar{I}_6^{a_2}(t))(1 - \lambda^{a_4}\bar{I}_6^{a_4}(t))^2 - q\Gamma_6^{a_4}\textstyle\prod_{k'=1}^{6}(1 - \lambda^{a_4}\bar{I}_{k'}^{a_4}(t))^{\psi(k',r)}.
\end{aligned}
\right.
$$

Here, $\Gamma_1^{a_4} =$
$1 - \frac{c(1-h)}{N_1^{a_4}}(F_2^{a_1a_3} + F_2^{a_2a_3} + F_3^{a_1a_3a_3} + F_3^{a_2a_3a_3} + F_3^{a_1a_2a_3} + F_4^{a_1a_2a_3a_3} + F_4^{a_2a_2a_3a_3})$,

$$\Gamma_2^{a_4} = 1 - \frac{2(1-c)(1-h)}{N_2^{a_4}}(F_2^{a_1a_3} + F_2^{a_2a_3} + F_3^{a_1a_3a_3} + F_3^{a_2a_3a_3} + F_3^{a_1a_2a_3} + F_4^{a_1a_2a_3a_3} +$$

$$F_4^{a_2a_2a_3a_3}) - \frac{F_2^{a_1a_4}}{N_2^{a_4}} - \frac{F_2^{a_2a_4}}{N_2^{a_4}},$$

$$\Gamma_3^{a_4} = \frac{F_3^{a_3a_3a_4} + 2F_3^{a_3a_4a_4}}{N_3^{a_4}},$$

$$\Gamma_4^{a_4} = \frac{F_4^{a_3a_3a_3a_4} + 2F_4^{a_3a_3a_4a_4}}{N_4^{a_4}},$$

$$\Gamma_5^{a_4} = \frac{F_5^{a_3a_3a_3a_3a_4} + 2F_5^{a_3a_3a_3a_4a_4} + 3F_5^{a_3a_3a_4a_4a_4}}{N_5^{a_4}},$$

$$\Gamma_6^{a_4} = q\frac{F_6^{a_2a_3a_3a_4a_4a_4}}{N_6^{a_4}},$$

and $\Gamma_k^{a_4}$ represents the proportion of individuals with household size-k, age group a_4 who do not have contact with minors during the daytime.
$\psi(k', r) = \left[r\frac{\Gamma_{k'}^{a_4} N_{k'}^{a_4}}{\sum_{k'} \Gamma_{k'}^{a_4} N_{k'}^{a_4}} \right]$ represents the number of individuals with household size-k', age group-a_4 among the r regular contacts in activity place.
